# Principal Components Analysis Based Unsupervised Feature Extraction Applied to Gene Expression Analysis of Blood from Dengue Haemorrhagic Fever Patients

**DOI:** 10.1038/srep44016

**Published:** 2017-03-09

**Authors:** Y-h. Taguchi

**Affiliations:** 1 Department of Physics, Chuo University, Tokyo, 112-8551, Japan

## Abstract

Dengue haemorrhagic fever (DHF) sometimes occurs after recovery from the disease caused by Dengue virus (DENV), and is often fatal. However, the mechanism of DHF has not been determined, possibly because no suitable methodologies are available to analyse this disease. Therefore, more innovative methods are required to analyse the gene expression profiles of DENV-infected patients. Principal components analysis (PCA)-based unsupervised feature extraction (FE) was applied to the gene expression profiles of DENV-infected patients, and an integrated analysis of two independent data sets identified 46 genes as critical for DHF progression. PCA using only these 46 genes rendered the two data sets highly consistent. The application of PCA to the 46 genes of an independent third data set successfully predicted the progression of DHF. A fourth *in vitro* data set confirmed the identification of the 46 genes. These 46 genes included interferon- and heme-biosynthesis-related genes. The former are enriched in binding sites for STAT1, STAT2, and IRF1, which are associated with DHF-promoting antibody-dependent enhancement, whereas the latter are considered to be related to the dysfunction of spliceosomes, which may mediate haemorrhage. These results are outcomes that other type of bioinformatic analysis could hardly achieve.

Dengue fever (DF) is a common mosquito-mediated infectious disease in tropical regions. Although it is typically non-fatal, it sometimes develops into life-threatening dengue haemorrhagic fever (DHF), which is associated with systemic haemorrhage^1^. Because DHF typically occurs after defervescence, DHF is not considered a symptom directly caused by the Dengue virus (DENV), which causes DF, but is thought to originate from the complex reaction of the host’s body to DF. However, how DHF develops from DF is not well understood. The exhaustive analysis of omics data is a useful strategy for resolving these kinds of problems, because a data-driven approach allows us to identify mechanisms that are difficult to predict with a rational knowledge-based discussion. Although it is not difficult to obtain various omics data for DF, they are not easy to analyse because they often include information for more than several tens of thousands of genes. In this case, the feature extraction (FE) and feature selection (FS) techniques are useful in determining what is happening within the data set obtained. FE tries to reconstruct a limited number of new features by combining given features, whereas FS tries to select a limited number of features from all the given features. The FE and FS techniques are divided into two categories: supervised and unsupervised. Most FSs are supervised and include huge numbers of implementations, ranging from simple FSs based on statistical tests between two classes^2^ to FSs that select a set of features based upon performance, e.g., random forest^3^. However, most FEs are unsupervised, including principal components analysis (PCA)^4^. Although some FEs are also supervised, such as partial least squares (PLS)^5^, unsupervised FS is rare because it is generally considered difficult to perform FS without any external criteria. However, if FS can be performed in an unsupervised way based upon a data-driven strategy, rather than in a supervised way based on some evaluation, e.g., classification performance or prediction accuracy, then it is possible that unsupervised FS could work better than supervised FS in some cases. For example, if samples are wrongly labelled, e.g., four classes are erroneous and only two classes are true, then supervised FS may select unappreciated features based upon the wrong classification, whereas unsupervised FS may not be misled by the non-existent four classes, because it is data driven. One of the problems of supervised FS is that it is not known whether all the labelling information is significantly related to the data set (observations) obtained.

There have been several trials of unsupervised FS. For example Ding^6^ proposed unsupervised FS for the analysis of gene expression based upon similarity. Li *et al*.^7^ performed FS using feature clustering. Wong *et al*.^8^ applied FS, based on consensus affinity, to microarray data. Unsupervised feature filtering (UFF)^9^ is based upon the entropy calculated on a leave-one-out basis. However, the analysis of all genes is computationally challenging. Ding^6^ used weights between all pairs of genes, whereas Li *et al*.^7^ and Wong *et al*.^8^ required the computation of the similarity between pairs of features. UFF requires the iterative computation of entropy, which computes entropy by removing features until a sufficiently small number of features remains. All of these methodologies require computational time proportional to the squared number of genes, which can be as many as tens of thousands, with iterative improvements.

Recently, PCA-based unsupervised FE^10^,^11^,^12^,^13^,^14^,^15^,^16^,^17^,^18^,^19^,^20^,^21^,^22^,^23^,^24^,^25^,^26^,^27^, which was initially proposed for the performance of PCA with selected features^27^, has been suggested for use in FS, especially in the integrated analysis of multiple (omics) data sets. PCA-based unsupervised FE requires the application of PCA to a gene expression matrix^10^/epigenetic profile^25^ only once. Therefore, it is not computationally challenging compared with previous unsupervised FSs and can be successfully applied to various gene selection problems. For example, the integrated analysis of promoter methylation in three distinct autoimmune diseases using PCA-based unsupervised FE identified the genes associated with aberrant promoter methylation that were common to the three diseases, which were identified by no other comparative method^26^. An integrated analysis of the mRNA/miRNA expression associated with posttraumatic stress disorder (PTSD)-mediated heart disease^18^ and various cancers^13^ identified a possible candidate gene associated with the diseases. An integrated analysis of gene expression and promoter methylation also successfully identified various disease-associated genes^11^,^14^,^15^,^19^. More recently, PCA-based unsupervised FE was used successfully in an integrated analysis of mRNA/miRNA expression and the metabolome^20^. In this paper, we applied PCA-based unsupervised FE to the mRNA expression profiles of DF and DHF patients and normal controls. An integrated analysis of two independent data sets allowed us to identify a limited number of possibly disease-associated genes, which was validated with an additional mRNA expression data set. The genes identified have been extensively shown to be associated with infectious viral diseases, suggesting the success of the methodology used here. We also propose a theoretical justification of this methodology, which works well for a wide range of FS/FE problems^11^,^12^,^13^,^14^,^15^,^16^,^17^,^18^,^19^,^20^,^21^,^22^,^23^,^24^,^25^,^26^,^27^, based upon a previously proposed theoretical framework^28^,^29^. Therefore, the purpose of this study was two-fold: to demonstrate the usefulness of PCA-based unsupervised FE and to propose a novel mechanism underlying DHF.

## Results

**Figure 1:**
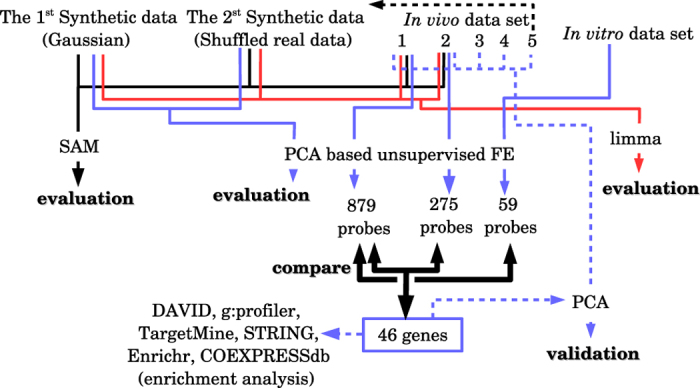
Overall flow chart. Thin black solid lines: data processing related to SAM. Red lines: data processing related to Limma. Solid blue lines: data processing related to PCA-based unsupervised FE. Broken blue lines: data processing related to (re-)embedding using the 46 selected genes. Bold black lines: data processing related to pairwise comparisons.

Figure 1 shows the overall flow of the analysis.

### Application of PCA-based unsupervised FE to synthetic examples

The use of a synthetic data set before the application of a methodology to a real data set is often useful in understanding the advantages and disadvantages of the proposed methodology. First, because we know the true answers for the synthetic data set (in contrast to the real data set), it is relatively easy to evaluate the performance of the methodology. Next, by preparing various data sets, we can intentionally generate a data set that can or cannot be successfully analysed with the proposed methodology, which allows us to understand the situations in which the proposed method is applicable. We can also demonstrate the superiority of the proposed method to conventional methods.

**Figure 2:**
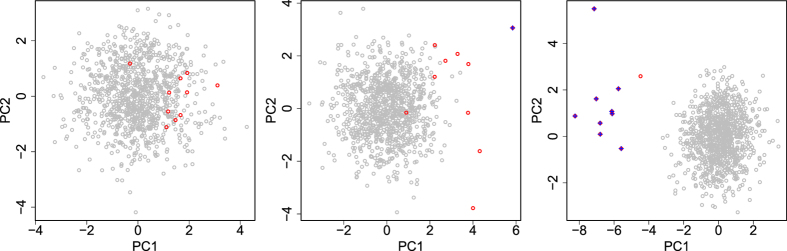
Scatter plots of the first and second PC scores, *u*_1i_ and *u*_2i_, attributed to genes of the first synthetic data set. Open grey circles represent the 990 genes not associated with the differential gene expression between the two classes, whereas the red open circles correspond to the 10 genes associated with the differential gene expression between the two classes. Blue crosses are those selected by PCA-based unsupervised FE. *s* = 1: top left, *s* = 1.5: middle and *s* = 2:right.

**Figure 3:**
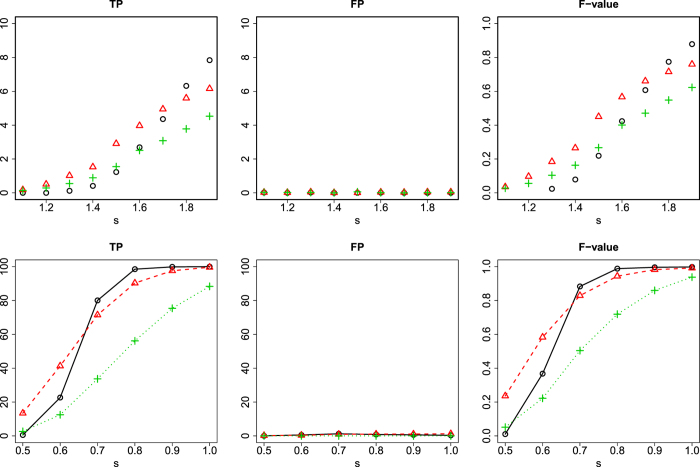
Various performances of PCA-based unsupervised FE applied to synthetic data. Upper row (the first synthetic data set, Gaussian): TP, FP, and F-measure for PCA-based unsupervised FE (open black circles), SAM correctly assuming two classes (open red triangles), and SAM wrongly assuming four classes (green crosses).Lower row (the second synthetic data set, compiled from real gene expression data): TP, FP, and F-measures for PCA-based unsupervised FE (open black circles), SAM correctly assuming two classes (open red triangles), and Limma (green crosses). Error bars (95% confidence interval) were less than the size of the characters.

To demonstrate how PCA-based unsupervised FE works and that it outperforms other popular FSs that are specifically designed for gene expression analyses, we compared PCA-based unsupervised FE with a significance analysis of microarrays (SAM)^30^ and Limma^31^ (see S1_Text for more details about how to perform SAM and Limma). The synthetically generated test data sets comprised “gene expression” data drawn from normal distributions, and the two classes to which each sample belonged had distinct or not distinct means (see Methods). Of 1000 genes, only the last 10 genes had expression patterns that differed between the two classes, whereas the expression of the first 990 genes did not, because it is reasonable to assume that in a real situation, the activities of a small number of genes are responsible for the observed phenotype(s). Each of the two classes included 10 samples, so in total, only 20 samples were considered. A small number of samples relative to the number of genes is also common in real experiments. The two classes had means of 0 and *s*(≥0). Therefore, a larger *s* indicates easier FS. *s* is also used as an enhancement factor for the expression of the 10 genes associated with the different gene expression patterns in the two samples, whereas the expression of the other genes is not enhanced. This also reflects the real situation: relevant genes should be more strongly expressed, whereas irrelevant genes should not be expressed. Figure 2 shows typical scatter plots of the PC scores attributed to 1000 genes when *s* = 1,1.5, or 2. Note that this poses a very difficult problem compared with the standard benchmark^32^, where the discrimination of the two classes is easier than in the present case because the difference between the two classes is greater. When *s* = 1, i.e., in the most difficult set-up, no genes were detected correctly, although no genes were wrongly identified. However, when *s* increased from 1 to 1.5, the number of correctly identified genes also increased to one of 10, and still no genes were wrongly identified. When *s* further increased to 2, nine genes were identified and no gene was wrongly identified. We performed averaging using 100 ensembles while changing *s* between 1 and 2. Figure 3 shows the dependence of true positives (TPs), false positives (FPs), and F-measures upon *s*. Here, in addition to TPs and FPs, we considered F-measures, which are useful performance measures for unbalanced data sets and are defined as [2(TP)/(FP + TP)⋅ (TP)/(TP + FN)]/[(TP)/(FP + TP) + (TP)/(TP + FN)], where FN represents false negatives. Although neither TP nor F-measure was large for smaller *s*, when *s* = 2, TP, FP, and F-measure had reasonable values. To compare the performance of this computation with that of SAM, we repeated the same computation using two SAM set-ups; one correctly assumed two classes, whereas the other wrongly assumed four classes (Fig. 3). Although the FPs obtained with both SAMs were small, the SAM that wrongly assumed four classes and the SAM that correctly assumed two classes were inferior to PCA-based unsupervised FE when *s* ≥ 1.6. and *s* ≥ 1.8, respectively. Although it was unsupervised, PCA-based unsupervised FE definitely outperformed SAM. Furthermore, although the performance of SAM decreased when four classes were wrongly assumed, PCA-based unsupervised FE circumvented this problem because it used no sample labelling information. Although we also tried to compare Limma, it did not identify any gene, including FPs, in this specific set-up, possibly because the parameter settings were too severe.

To perform comparisons with more-realistic synthetic data sets, we generated a gene expression data set composed of two classes using one set of the DENV gene expression profiles (data set 5, see Methods) analysed in this study. After the expression of each gene was standardized, the samples were divided into two classes, each containing half the samples. The positive constant *s* was added to the samples in one of the two classes such that the two classes were distinct. Therefore, a larger *s* also indicates an easier resolution of the problem. Figure 3 shows the results averaged using 100 ensembles while *s* was changed from 0.5 to 1. The overall performance achieved was relatively similar to that achieved with the first synthetic data set. PCA-based unsupervised FE again outperformed the other methodologies only for larger *s (s* ≥ 0.7), although Limma identified non-negative TPs with smaller values in this second synthetic data set than were identified with the other two methods.

Although we can conclude from its application to the two synthetic data sets that PCA-based unsupervised FE outperforms two popular FSs proposed for the analysis of “gene expression” data when *s* ≥ 1.8 (for the first synthetic data set) or *s* ≥ 0.7 (for the second synthetic data set), it is unclear whether PCA-based unsupervised FE would outperform these two methods when the set-ups were further modified. In fact, there is no way to check the superiority of PCA-based unsupervised FE to these two methods in all possible situations. Therefore, comparisons made with real examples are required.

The theoretical background and further advantages of this methodology are discussed in S1_Text.

### Application of PCA-based unsupervised FE to gene expression in DENV-infected patients

To demonstrate the utility of PCA-based unsupervised FE when applied to real data sets and to understand how DF progresses to DHF based upon a gene expression analysis, we used this method to analyse the gene expression patterns of multiple DENV-infected patients. We used multiple gene expression profiles because the comparison and integration of multiple profiles allows us to identify more-robust platform-independent outcomes.

The first example (data set 1, GSE51808) was obtained by Kwissa *et al*.^33^. It includes four categories: DHF patients, DF patients, convalescent patients (CP), and healthy controls (HC). When investigating the PC loadings that differ between the groups, we found that PC2 (with a contribution of only 1.45%) and PC3 (with a contribution of only 0.45%) differed between DHF + DF and CP + HC; the *P*-values computed with a *t* test rejected the null hypothesis that the mean *v*_*kj*_ within DHF + DF and the mean within CP + HC were identical in favour of the hypothesis that they were not: 1.03 × 10^−21^ for PC2 and 4.56 × 10^−3^ for PC3. Although the contribution of the first PC was 95.5%, it did not differ significantly among the four classes. Figure S1 in S1_Text shows a biplot of PC1 to PC3, where PC1 clearly displays no sample dependence (the first PC loading attributed to all samples has the same value). However, it is obvious that DHF + DF and CP + HC are well separated in the two-dimensional space spanned by the PC2 and PC3 loadings. This suggests that PCA-based unsupervised FE correctly identifies the space in which DHF + DF and CP + HC are well separated. On this plane, we selected 879 probes as outliers (see sheet 4 in S1_File for the full list of genes associated with the 879 probes). Because 879 probes were too many to be considered critical to DHF and to establish a smaller and more reliable set of genes, we applied PCA-based unsupervised FE to a second data set (data set 2, GSE13052^34^) to further screen the genes. Figure S1 in S1_Text also shows the biplot of PC1 to PC3, where PC1 again clearly displays no sample dependence (again, the first PC loadings attributed to all samples have the same values). PC2 and PC3 were again selected as the PCs used for FE. The contributions of PC2 and PC3 were only 3.39% and 2.90%, respectively. The *P*-values computed with a *t* test that rejected the null hypothesis that the means *v*_*kj*_ within the convalescent and acute patients are identical in favour of the hypothesis that where they were not were 1.65 × 10^−5^ for PC2 and 7.15 × 10^−3^ for PC3. Although the contribution of the first PC was 89.5%, it did not differ significantly among the four classes (see Fig. S1 in S1 Text). However, the convalescent and acute patients were well separated in the two-dimensional space spanned by the PC2 and PC3 loadings. This suggests that PCA-based unsupervised FE correctly identified the space in which the convalescent and acute patients were well separated. On this plane, we selected 275 probes as outliers (see sheet 4 in S1_File for the full list of genes associated with the 275 probes). We identified the 46 common genes that were common to the 879 and 275 genes identified with the first and the second data sets (data sets 1 and 2), respectively (Table 1). These are expected to be more robust and more reliable than the genes identified in either data set alone because they were detected in two data sets using different platforms.

To confirm that the expression of these 46 genes did actually differ between the healthy controls and patients, we performed a clustering analysis of the samples in data sets 1 and 2 using only these 46 genes. Figure S3 in S1_Text shows the heatmaps produced. It is clear that the symptomatic patients (with fever) are well separated from both the healthy controls and the patients without symptoms. This suggests that PCA-based unsupervised FE successfully identified a limited number of genes that discriminate the two groups well.

The fact that gene expression can distinguish patients with symptoms from healthy controls, but cannot distinguish patients with DF from those with DHF is consistent with the heatmap produced with all genes by Kwissa *et al*.^33^. Selecting a limited number of genes to reproduce the results using all genes is not straightforward. Genes are usually selected based on their differential expression. However, to do this, we must decide the kind of difference to be considered. For example, if we select genes based upon their differential expression between DF and DHF, the outcome may differ from that when we use all the genes that cannot be used to distinguish DF and DHF. Therefore, reducing the number of genes is highly context dependent. In contrast to this, our unsupervised approach can identify a limited number of genes that produce the outcome produced using all genes, because we need no criterion based upon sample labelling or classification. Despite this, the 46 genes selected with our methodology reproduced the outcome obtained using all genes, which demonstrates the superiority of our methodology.

**Figure 4:**
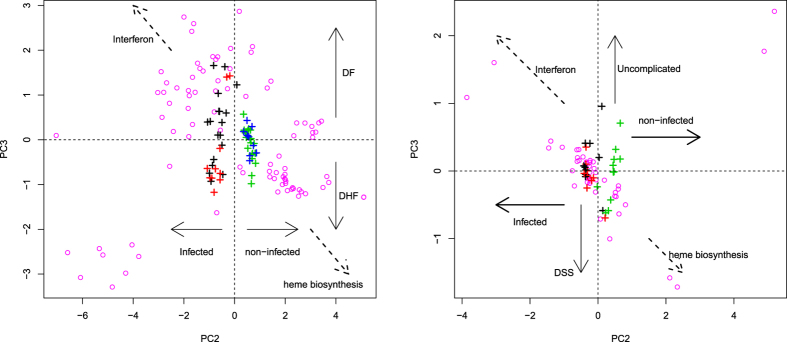
Biplots of PC2 and PC3 scores, *u*_2i_ and *u*_3i_, computed using only the 46 genes selected with PCA-based unsupervised FE. Left: data set 1 (GSE51808); right: data set 2 (GSE13052). Open magenta circles are PC scores for the probes associated with the 46 genes (for more details on the biological features of individual genes, see the enrichment analysis available in sheets 1–3 in S1_File). The contributions of PC2 and PC3 increased to 13% and 4% (left: data set 1), respectively, and to 7% and 1.6% (right: data set 2), respectively. Black (red) crosses represent PC loadings, *v*_2*j*_ and *v*_3*j*_, of the DF (DHF) patients. Blue crosses correspond to healthy controls. Green crosses represent convalescent patients. Broken arrows show the different gene functions (see sheets 1-3 in S1_File). DSS, Dengue shock syndrome.

To confirm that we had successfully selected critical genes representing the relationships between samples, we applied PCA to *x*_*ij*_s using only the probes associated with the 46 selected genes (Therefore, this is not only FS but also FE). That 46 genes alone can represent disease progression suggests the reliability of our methodology and the biological interpretation that can be drawn from the analysis of these 46 genes. Figure 4 shows the results. PC2 and PC3 were again selected to draw the biplot and the PCs were more easily interpreted. PC2 represented the distinction between patients that display symptoms (i.e., fever) and those that do not (i.e., healthy controls and convalescent patients). PC3 represents the distinction between DHF (dengue shock syndrome [DSS]) and DF (uncomplicated). Remarkably, using the 46 identified genes, the scatter plots of the PC loadings (samples) for data sets 1 and 2 became common. The samples were aligned beside PC3 on both sides of the origin. Infected patients were roughly divided into the upper and lower half, which corresponded to DF and DHF, respectively. More interestingly, the scatter plots of the PC scores (genes) correlated significantly between data sets 1 and 2 (Fig. S4 in S1_Text). These common embedding structures of the samples, as well as those of the genes in data sets 1 and 2 shown in Fig. S4 in S1_Text, demonstrate the robustness of PCA-based unsupervised FE and the applicability of this methodology.

**Figure 5:**
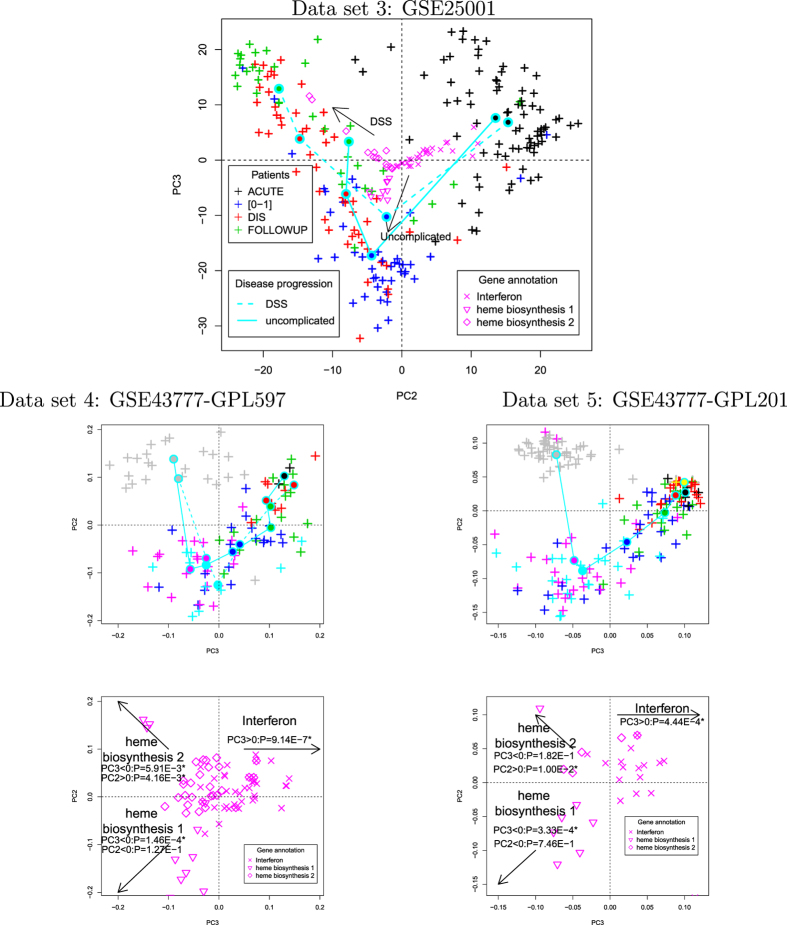
Biplots for data sets 3 to 5. Top: Biplot of data set 3 (GSE25001) using only the 46 genes identified in data sets 1 and 2. Open magenta symbols ×, ∇, ◇ are the PC scores, *u*_2*i*_ and *u*_3*i*_, for the probes associated with the 46 genes (for more details on the biological features of individual genes, see the enrichment analysis available in sheets 1–3 in S1_File). The contributions of PC2 and PC3 increased to 11% and 6.3%, respectively. Crosses (+) represent the PC loadings, *v*_2*j*_ and *v*_3*j*_, of the patients. Filled circles connected by solid cyan lines represent the centres of mass for each group (see legends for more details). Middle left: Scatter plot of the PC scores for data set 4 (GSE43777-GPL507) using 76 probes attributed to any of the 46 genes. The contributions of PC2 and PC3 increased to 5.3% and 4.0%, respectively. The correspondence between the coloured crosses (+) and disease progression are black (stage G1), red (stage G2), green (stage G3), blue (stage G4), cyan (stage G5), magenta (stage G6), and grey (stage G7). Cyan solid and broken lines correspond to DF and DHF, respectively. Middle right: Scatter plot of the PC scores in data set 5 (GSE43777-GPL201) using 28 probes attributed to any of the 46 genes. The contributions of PC2 and PC3 increased to 14% and 10.0%, respectively. The correspondence between the coloured + symbols and disease progressions are the same as for GSE43777-GPL507 and yellow (stage G0). Solid cyan line corresponds to patients with either DF or DHF. Bottom: Scatter plots of PC loading (left to right: GSE43777-GPL507/201). *P*-values were computed with a *t* test to determine whether the PC scores had positive (or negative) mean values.

Although PCA-based unsupervised FE identified essential genes and common biological structures in two independent data sets, it is still possible that this was an a coincidence. To test this hypothesis, we applied PCA-based unsupervised FE to data set 3 (GSE25001^35^). If the 46 genes selected also describe disease progression in another additional data set, our conclusions will be strengthened. Figure S1 in S1_Text shows a scatter plot of the PC scores attributed to the probes. Again PC2 (contribution 5.3%) and PC3 (contribution 1.4%) are shown. In this embedding, the 46 genes selected in both data sets 1 and 2 form a trigram Y shape, meaning that the 46 genes are grouped into three categories, each of which shares the same sample dependence. This Y shape is unlikely to be an accidental coincidence because data set 3 was not used to select the 46 genes. Finally, only 46 genes were embedded by the PCA (Fig. 5). Although data set 3 was not used to identify the 46 genes, the timescale of DF/DHF development is well represented. In the early stage of infection, there were no significant differences between DF and DHF. With increasing time, the distinction between DF and DHF increased and was largest in the follow-up stage (i.e., after recovery). To investigate this quantitatively, we applied a *t* test to the second and third PC scores between the “DSS” and “uncomplicated” groups (Table 2). It is obvious that only in the disease (DIS) stage and follow-up (FOLLOWUP) stage do the PC scores differ between the “DSS” and “uncomplicated” groups. This confirms that we have successfully identified, using PCA-based unsupervised FE, the 46 genes in data sets 1 and 2 that represent DHF/DF progression, even in the independent third data set.

Why did we specifically select these three gene expression profiles? This study was fully data driven, and with a data-driven approach, we try to integrate multiple data sets that may generate reliable outcomes, like those we used in the present study. Because our approach was successful, the selection of these data sets was also successful.

### Comparison with other supervised and unsupervised methodologies

Although we have demonstrated the usefulness of our unsupervised methodology, we should explain why we did not use other popular supervised methods but intentionally used an unsupervised method, because it is generally supposed that supervised FE outperforms unsupervised FE. To demonstrate the superiority of our unsupervised FE over other frequently used supervised methods, we compared our methodology with SAM and Limma, two major implementations of FE specifically adapted for gene expression analyses. Using SAM and Limma and assuming two or four classes, we identified genes associated with adjusted *P*-values of less than 0.01 (Table S2 in S1_Text). However, when the same “adjusted *P*-values less than 0.01” criterion was applied, too many genes were identified in both sets to identify the intersections between data sets 1 and 2, as was done with PCA-based unsupervised FE. Although it might be possible to filter the genes further using additional criteria, e.g., the fold change, it is obvious that these two methodologies are inferior to PCA-based unsupervised FE, because PCA-based unsupervised FE requires no criteria other than *P*-values. How well would the other above-mentioned unsupervised FSs^6^,^7^,^8^,^9^ perform when applied to the present data sets? In the gene expression profiles analysed in this study, the number of genes exceeded a few tens of thousands, and the above-mentioned unsupervised FSs, other than Ding’s study^6^, would entail computational complexities proportional to the square of the number of genes. Therefore, it is unrealistic to apply these methods to the present data sets. Consequently, we could not compare our performance with that achieved with the above-mentioned unsupervised FSs. The methodology reported in the study by Ding^6^ is very similar to our methodology. Ding^6^ ordered genes based upon a two-way ordering system, assuming a gene expression matrix as the bipartite graph, and discarded the middle-ranked genes. However, the genes themselves were not ranked based upon PC scores other than the first one obtained with PCA, and it did not outperform a simple supervised method according to a *t* test in his trials. Ding also stated clearly that gene expression must be non-negative in this implementation because the gene expression matrix must be treated as weights in the bipartite graphs. Therefore, he could not apply scaling such that ∑_*i*_*x*_*ij*_ = 0 and 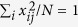 to the gene expression as we have done. Ding^6^ came close to the idea presented in this study, but missed the central point: not samples but features (genes) should be embedded and PC scores other than the first score should be considered for FSs, even if their contributions are apparently very small.

## Discussion

We investigated the robustness and biological significance of the 46 genes identified.

First, because the results obtained may have been accidental, we used additional data sets to determine whether the selection of genes with PCA-based unsupervised FE was robust. We used an *in vitro* study to enhance the robustness of the results, because the data sets analysed were from an *in vivo* study. If the genes selected are consistent with those selected in the *in vitro* study, the outcome is more trustworthy. Interestingly, the 46 genes included many genes previously reported to be associated with DENV in *in vitro* studies^36^, i.e., *CD38*, *HERC5*, *IFI44L*, *IFIT3*, *LY6E*, *OA*, *OASL*, *RSAD2*, *TRAIL*, (*TNFSF10*), and the anti-viral activity of *TRAIL* against DENV has been confirmed experimentally^36^. Furthermore, after applying PCA-based unsupervised FE to the data set analysed by Warke *et al*.^36^, we found that 59 probes were associated with aberrant gene expression between the control and DENV-infected cell lines (see sheet 4 in S1_File for the full list of genes associated with the 59 probes). Among the genes associated with these 59 probes, the genes shared with those identified in the present study were identified: *APOBEC3A*, *IFI27*, *IFI35*, *IFIT2*, *IL1RN*, *ISG15*, *MX1*, and *OSA1*. Thus, 17 of the 46 genes were also detected in an *in vitro* study. Moreover, 10 of the 46 genes were recognized as anti-viral interferon-stimulated genes (ISG)^37^, whereas *APOBEC3*, *IFI44L*, *IFIT2/3*, *ISG15*, *MX1*, *OAS1/3/L*, and *RSAD2. CD38* are reported to be associated with immune thrombocytopenia^38^.

Second, we used two additional *in vivo* gene expression profiles (data sets 4 and 5, GSE43777, see methods) also associated with disease progression during eight distinctive stages, G0 to G7. Because Sun *et al*.^39^ identified a triangular configuration in their two-dimensional PCA embedding, their data set was suitable for testing the robustness of our 46 genes (for a more detailed comparison with their results, see below). Basically, these genes reproduced the configuration of data set 3, although with some differences (Fig. 5). First, the time progression in data sets 4 and 5 on the plane spanned by PC2 and PC3 was V-shaped, which was also seen in data set 3. PC3 is the vertical axis in data set 3, whereas PC2 is the vertical axis in data sets 4 and 5. However, because the order of the PCs is simply dependent on their contributions, their order is not biologically important if the overall configuration is conserved. Finally, although the Y-shaped configuration of the genes observed in data set 3 is missing from data sets 4 and 5, the overall configuration is similar. Interferon and heme biosynthesis 1 and 2, are located in the right half plane, the second quadrant, and third quadrant of data sets 3, 4, and 5, respectively. This is obvious in data set 3, although a *t* test was also used to determine whether the PC scores had negative or positive mean values in data sets 4 and 5 in order to determine the quadrant in which the center of the genes was located (see captions). Therefore, the configuration of the PC scores in data set 3 observed in Fig. 5 is expected to be robust. After a *t* test was applied, the distinction between DHF and DF in the follow-up samples seen in data set 3 was still observed between DHF and HF at stage G7 in data set 4. The 2nd PC loading attributed to DF patients was significantly larger than that of the DHF patients (*P* = 0.05 and 0.04 with a *t* test and Wilcoxon signed-rank sum test, respectively). Because data set 5 is predominantly composed of DF only, we did not check this point in data set 5. The relatively weak distinction between DF and DHF in data set 4 may be because these samples were collected at different times (data set 3: FOLLOWUP, ≥72h after illness onset; data set 4: G7 (convalescent) samples, around day 28 after the first sampling). In fact, there was a large gap between the G7 samples (grey +) and the G6 (late acute) samples (magenta+) for data sets 4 and 5 (middle panels of Fig. 5). It is possible that the uncollected samples reflect the distinction between DF and DHF. We require more samples to confirm this point.

Because we successfully confirmed the robustness of our results, we next investigated the biological reliability of the 46 selected genes. We uploaded the 46 genes to three enrichment analysis servers, DAVID^40^, g:pfofiler^41^ and TargetMine^42^ (see sheets 1–3 in S1_File for the full list of enriched biological terms and pathways), to compensate for the bias introduced by each individual enrichment server. g:Profiler reported the enhancement of the IRF/IRF-7 motif, which is known to occur in interferon (IFN)-related transcription factors (TFs)^43^. Both g:Profiler and TargetMine also detected the measles and influenza A pathways (hsa05162 and hsa05164, respectively). Measles and influenza are often listed as diseases that differ negligibly from DF in terms of their diagnosis^44^,^45^. There are no DENV pathways in the Kyoto Encyclopedia of Genes and Genomes (KEGG) database. Therefore, it is reasonable that these two viruses were detected instead of DENV. Other than these, multiple enrichments related to either viral infection or haemorrhage were detected. For example, Gene Ontology (GO) biological process (BP) terms GO:0009615 (response to virus), GO:0006955 (immune response), and GO:0015671 (oxygen transport) were identified by all three servers. GO cellular component (CC) term GO:0005833 (haemoglobin complex) was also identified by all three servers. Reactome pathways REAC:913531 (interferon signalling), REAC:909733 (interferon alpha/beta signalling), REAC:168256 (immune system), and REAC:1280215 (cytokine signalling in immune system) were identified by g:Profiler and TargetMine. Further haemorrhage-related or viral-infection-related GO BP terms were detected by g:Profiler and TargetMine.

The next step in the biological validation process was to determine the interactions between these genes. If they have tight relationships, the selection of the 46 genes is more reliable because single proteins rarely function without the collaboration with other proteins. To check this, we uploaded the 46 genes to the STRING server^46^, which detected 96 protein–protein interactions among the products of the 46 genes (*P* = 0 within the numerical accuracy), although the expected number of protein–protein interactions was only eight. Therefore, the 46 identified genes were also enriched for protein–protein interactions, probably because of the functional collaborations between the products of these genes.

These analyses suggest that PCA-based unsupervised FE can successfully identify a biologically feasible set of genes.

**Figure 6:**
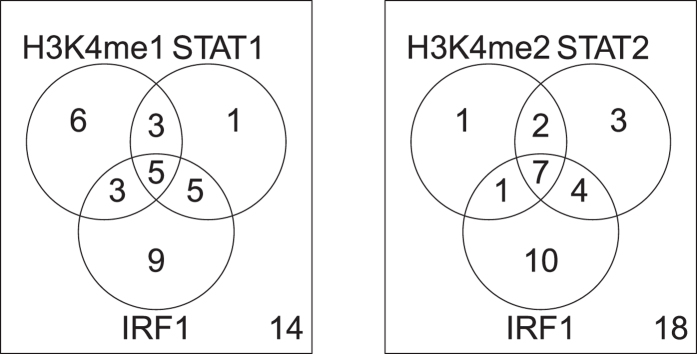
Venn diagrams of the enrichment of TF-binding sites and histone modifications in the 46 genes in the K562 cell line, identified with Enrichr. These clearly overlap strongly. Left: STAT1_K562_hg19, IRF1_K562_hg19 (ENCODE TF ChIP-seq 2015), and H3K4me1_K562_hg19 (ENCODE Histone Modifications 2015); right: STAT2_K562_hg19, IRF1_K562_hg19 (ENCODE TF ChIP-seq 2015), and H3K4me2_K562_hg19 (ENCODE Histone Modifications 2015).

To further investigate the biological backgrounds of the 46 selected genes, we uploaded the 46 genes to Enrichr^47^, a multi-functional enrichment analysis server. Among the results given by Enrichr, we noticed the top-ranked three transcription factor (TF) bindings at “ENCODE TF ChIP-seq 2015”, STAT1, STAT2, and IRF1 in K562 cells. K562 is a cell line often used in *in vitro* DENV infection experiments (see references cited below). The 46 genes were also enriched for multiple histone modifications (Table 3). The genes associated with histone modification largely overlapped the TF target genes (Fig. 6). Ni *et al*.^48^ reported that the biphasic formation of a STAT1/IRF1 complex is accompanied by histone methylation, which is consistent with the observed enrichment of STAT1- and IRF1-binding sites in these 46 genes. More interestingly, Schoggins *et al*.^49^ identified STAT2 and IRF1 as effective inhibitors of DENV infection. Many studies have also reported the cooperation between these TFs. Kumatori *et al*.^50^ reported that STAT1 and IRF1 cooperatively regulate the expression of the *GP91* gene. Wang *et al*.^51^ reported that the STAT1/IRF-1 signalling pathway mediates the injurious effects of IFN-gamma on oligodendrocyte progenitor cells. Therefore, identifying the enrichment of these three TFs is unlikely to be accidental. We also found that antibody-dependent enhancement (ADE) is a factor potentially involved in the direct relationship between DF and these TFs. Chareonsirisuthigul *et al*.^52^ showed that the ADE infection pathway suppresses the innate anti-DENV mediator, the nitric oxide (NO) radical, by disrupting the transcription of the inducible nitric oxide synthase (iNOS) gene by TFIRF1, and blocking the activation of STAT1. ADE is believed to be a potential cause of DHF because DENV-ADE infection has a greater effect on viral replication than DENV infection^53^,^54^. In contrast, Huang *et al*.^55^ reported that neither DENV infection nor ADE-DENV infection upregulates IL10 or IL6 expression, and these proteins were not encoded by any of the 46 genes identified in the present study. As can be seen in Fig. 5, because the PCA of these 46 genes not only described the DF-to-DHF progression, but also distinguished between DF and DHF, these genes must include those responsible for DHF. Therefore, detecting the enrichment of these TF-bound genes may be the key to distinguishing between DHF and DF. This suggests that among our 46 selected genes, the genes targeted by these three TFs are expressed downstream from STAT1, STAT2, and IRF1, and are the ADE targets among the 46 selected genes. To our knowledge, this is the first report to identify the genes downstream from ADE-DENV using a bioinformatic (meta) analysis rather than experiments.

Although we hypothesize that our 46 selected genes include the genes associated with ADE, this only refers to immunology-related genes. As can be seen in Fig. 5, the 46 genes also include many heme biosynthesis genes. To determine the relationship between DHF and these heme biosynthesis genes, we uploaded the genes to another data analysis server, COEXPRESSdb^56^, which infers a possible set of genes within the list of uploaded genes that might be co-expressed. In this way, we found 11 spliceosome (hsa03040)-related genes among the 46 genes, which might be co-expressed (see Figs S8 and S9 in S1_Text and sheet 5 in S1_File). Interestingly, Hess *et al*.^57^ have already reported that U-spliceosomal non-coding RNAs (ncRNAs) are affected during DENV infection. Pre-mRNA splicing not only occurs in the cytoplasm of platelets, but also provides a mechanism for regulating cytokine production after platelet activation^58^, which is related to the innate immune response^59^. It is clear that reduced platelet counts might lead to a severe bleeding diathesis. This suggests that the dysfunction of spliceosomes causes haemorrhage, and is, to the best of our knowledge, the first such proposal. Further and follow-up studies are required.

We also compared our results with those of the studies from which the data sets used in this study were taken. Why didn’t we compare the genes we selected with those selected in the original studies? 33,34,35,39. Neither Kwissa *et al*.^33^ nor Hoang *et al*.^35^ provided a list of the selected genes, possibly because they could not select a sufficiently small number of genes to investigate them individually in detail. Long *et al*.^34^ selected 100 genes for each of the three pairwise comparisons (DHF vs DF, CP vs DF, and CP vs DHF), 300 genes in total, similar to the number we selected (275 genes). Thus, the only comparison possible is between our 275 genes and their 300 genes. When we replaced our 275 genes with the 300 genes selected by Long *et al*. in data set 2, only 83 genes were selected from both data sets 1 and 2 (see sheet 6 in S1_file). Because 83 genes are more than our 46 genes, Long *et al*. apparently identified more coincidental results between data sets 1 and 2 than did our analysis. However, when both the 83 genes and the 46 genes were uploaded simultaneously to g:profiler, the impression was reversed. The 83 genes are primarily enriched in genes involved in the cell division cycle, which is unlikely to be related to DF (see the enrichment analysis in S1_Text). This suggests that our methodology identified a more limited but more biologically reliable set of genes than did that of Long *et al*. Among the five studies from which the gene expression profiles were taken, the study by Sun *et al*.^39^ was most similar to ours. Those researchers even embedded samples in two-dimensional space with PCA, using only the genes that they selected and that formed the triangular configuration seen in our genes in Fig. 5. However, our results still have multiple advantages over theirs. First, they selected 313 genes, which is over six time more than the number we selected (46 genes). Because they selected genes for each of the eight disease stages (G0 to G7) and there were very few overlaps between them, they could not identify a restricted number of genes that represented the overall disease progression. Their PCA embedding of samples was also less consistent with disease progression than ours. Therefore, they had to merge the eight stages into three groups, the early acute, late acute, and convalescent groups, to obtain biologically interpretable results. Furthermore, only some (23) of our 46 genes were included in their 313 genes. Fifteen and 13 of our genes were included in the genes detected in the early and late acute stages, respectively, and only five of our genes were included among the genes detected in both the early and late acute stages. Therefore, our methodology identified a more reliable, smaller, more robust, and distinct set of genes than the analysis of Sun *et al*.

Throughout the manuscript, we have almost always argued that the identification of too many significant genes is neither trustworthy nor usable. There can be two objections to this opinion:

1. It is always possible to reduce the number of genes by using a smaller number of top-ranked (relatively more significant) genes. Therefore, the identification of too many significant genes is not a problem at all.
2. The identification of too many significant genes may be evidence that the methodology works even when smaller samples are considered (because generally smaller samples induce larger, less significant *P*-values).

Objection 1 is meaningless from a statistical perspective because adjusted *P*-values are usually regarded as a portion of FP. This means that requiring very small adjusted *P*-vales (e.g., less than 1/*N*) does not make sense. Therefore, selecting the top-ranked genes does not guarantee more-reliable results. To demonstrate this, we used Limma or SAM to analyse the top-ranked 879 or 275 genes, which were the numbers identified with PCA-based unsupervised FE from data sets 1 and 2, respectively, and counted the overlaps between them (there were 46 overlaps when PCA-based unsupervised FE was used). We found that only one and two genes were commonly selected when Limma was used for the two- and four-class classifications, respectively. For SAM, because more than a thousand genes were associated with adjusted *P*-vales equal to zero in data set 1 for both the two-class and four-class classifications, we could not even select a smaller number of significant genes. This definitely suggests that simply taking the top-ranked genes without statistical reliability is not an appropriate alternative to a method that can select a reliable number of genes based on a statistically meaningful assumption (e.g., adjusted *P*-values less than 0.01). Thus, objection 1 is refuted, at least for our case. As for objection 2, we tried to select genes using samples containing half the data from data sets 1 and 2, using either SAM, Limma, or PCA-based unsupervised FE (Table S2). The results for SAM and Limma were very disappointing. The number of genes selected often remained unchanged (i.e., too many) or became too small (even less than 1). This suggests that objection 2 holds only for very specific combinations of methods and sample numbers, so it is not worthy of consideration. In contrast, PCA-based unsupervised FE selected almost the same number of genes independently of the sample numbers. This may seem strange. However, PCA-based unsupervised FE selects genes based upon the PC scores attributed to individual genes. PCA is essentially a projection from a high dimension to low (two) dimensions. In this context, halving the sample numbers corresponds to the random elimination of half the high-dimensional space, which is unlikely to change the projection dramatically. If the PC scores are not altered much, the genes selected remain unchanged, even after the sample numbers are halved. For these reasons, we conclude that objections 1 and 2 need not be considered, at least in the present study.

Finally, we will briefly discuss the relationship between the robustness of our methodology and the heterogeneity of the data sets. Our results may be considered untrustworthy because we derived most of our conclusions from the study of heterogeneous data sets collected from multiple studies that had distinct experimental plans, used different platforms (microarrays), and had distinct purposes. How can we derive something valid from such diverse data sets? Our methodology, PCA-based unsupervised FE, is known to generate robust results from an integrated analysis of heterogeneous data sets. For example, we previously analysed mouse cardiac maturation based on data sets collected with two distinct microarray platforms^60^. The selected genes were biologically useful when they were considered in human samples. This process is similar to that used in the present study, in which the integration of data sets 1 and 2 identified a gene set that described disease progression in data set 3. Alternatively, in an integrated analysis of mRNA and miRNA, we successfully compared the mRNA and miRNA expression measured in different studies^13^. All these studies suggest that our methodology is sufficiently robust to derive biologically reliable outcomes, even from heterogeneous data sets. Contrary to the impression that the use of heterogeneous data sets is erroneous, it can provide rather robust and reliable results if a suitable methodology is used. It even strengthens the robustness of the outcomes if it is successful, because although the use of heterogeneous data sets has greater potential for failure, the conclusions are less likely to be accidentally reliable while actually being untrue. Today, although RNA-seq technology is superceding microarrays because they are less reliable, if suitable methods such as PCA-based unsupervised FE are used, suitable results can still be obtained. In fact, PCA-based unsupervised FE has also been used in an integrated analysis of microarray and RNA-seq measurements^10^,^20^.

## Methods

### Synthetic data set

#### First synthetic data set

In the first synthetic gene expression data set (data set 1), the gene expression values were uncorrelated random numbers drawn from a Gaussian distribution with common variance. The number of genes was assumed to be much larger than the number of samples. The samples formed two classes, each of which included the same number of samples, but they differed only for very small numbers of genes among all genes. The mean value within each class was taken to be different, so that they were discriminative. The majority of the remaining genes did not form two classes. The mathematical formulation was as follows.

To simulate gene expression with *N* genes and *M* samples, *x*_*ij*_s (*i* = 1, …, *N*, *j* = 1, …, *M*) were drawn from the normal distribution 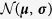, where ***μ*** ≥ 0 is the mean and *σ* > 0 is the standard deviation, and


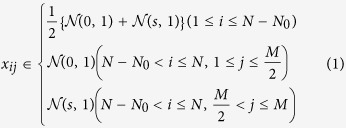


Then, *x*_*ij*_s (*N* − *N*_0_ < *i* ≤ *N*) are enhanced by factor *s* as follows: *x*_*ij*_ ← *s* × *x*_*ij*_. In this study, *N* = 1000 and *N*_0_ = 10.

#### Second synthetic data set

Because the gene expression data drawn from a Gaussian distribution are unlikely to be similar to real data sets, we compiled a more realistic but synthetic data set from data set 5 (see below). Gene expression was standardized to have a zero mean and a variance of one for each gene. The samples were then shuffled within each sample. The shuffled samples were divided into two classes. Some positive constant was added to only the samples within one of the two classes. The mathematical formulation is as follows.

Suppose *x*_*ij*_ is the gene expression of the *i*th gene of the *j*th sample that belongs to data set 5. To standardize gene expression, *x*_*ij*_ was converted to


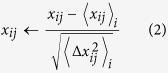



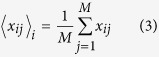



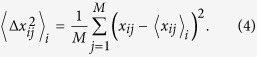


Then *x*_*ij*_ was shuffled within each gene as


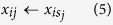


where *s*_*j*_ is a random integer drawn from (1, …, *M*) without replacement. *s*_*j*_ is independently drawn for each *i*. The positive constant *s* was then added to the first *N*_0_ genes in the first half of the samples,


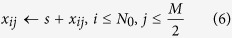


In data set 5, *N* = 8793, *M* = 168 and *N*_0_ is taken to be 100.

### PCA-based unsupervised FE

#### PCA

In contrast to the usual use of PCA, where samples are embedded, the genes were embedded in this implementation.

Suppose *x*_*ij*_s satisfies 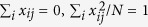 and *X* is a matrix whose elements are *x*_*ij*_. The gram matrix *G* is defined as *G* ≡ *XX*^*T*^. Eigen vectors **u**_*k*_ = (*u*_*k*1_, …, *u*_*kN*_)^*T*^s (1 ≤ *k* ≤ *min(M*, *N*)) are then obtained as *G***u**_*k*_ = *λ*_*k*_**u**_*k*_, where *u*_*ki*_ is the *k*th PC score attributed to gene *i* and *λ*_*k*_s are the Eigen values ordered as *λ*_*k*_ ≥ *λ*_*k*_ + 1. The *k*th PC loadings attributed to the *j*th sample **v**_*kj*_ are defined as **v**_*k*_ = *X*^*T*^**u**_*k*_, where *v*_*k*_ = (*v*_*k*1_, …, *v*_*kM*_)^*T*^ because **v**_*k*_ is the Eigen vector of the covariance matrix *X*^*T*^*X*, *X*^*T*^*G***u**_*k*_ = *X*^*T*^*XX*^*T*^**u**_*k*_ = *X*^*T*^*X***v**_*k*_ = *λ*_*k*_*X*^*T*^**u**_*k*_ = *λ*_*k*_**v**_*k*_.

#### PCA-based unsupervised FE applied to the synthetic data set

First, we computed the *P*-values that rejected the null hypothesis that the mean of {*v*_*kj*_|*j* = 1, …, (*M*)/(2)} is equal to that of {*v*_*kj*_|*j* = (*M*)/(2) + 1, …, *M*} in favour of the alternative hypothesis, that the mean of {*v*_*kj*_|*j* = 1, …, (*M*)/(2)} is not equal to that of {*v*_*kj*_|*j* = (*M*)/(2) + 1, …, *M*}. The *k*′th PC associated with the smallest *P*-value was then selected and used for FE, as a smaller *P*-value corresponds to a more significant difference between {*v*_*kj*_|*j* = (*M*)/(2) + 1, …, *M*} and {*v*_*kj*_|*j* = 1, …, (*M*)/(2)}. Assuming that *u*_*k*′*i*_ obeys a normal distribution, *P*-values were then attributed to the *i*th gene using the *χ* squared distribution. The *P*-values were further adjusted by the Benjamini–Hochberg (BH) criterion^61^, and the genes associated with adjusted *P*-vales less than 0.01 were selected as the genes associated with the difference between 1 ≤ *j* ≤ (*M*)/(2) and (*M*)/(2) < *j* ≤ *M*.

#### PCA-based unsupervised FE applied to gene expression in DENV patients

We identified a set of *k*s, {*k*}_*sig*_, associated with *P*-values less than 0.05 that rejected the null hypothesis that the means of the PC loadings (*v*_*kj*_) were identical over multiple classes, in favour of the alternative hypothesis that the means were not identical over multiple classes. Assuming that *u*_*k*′*i*_s are normally distributed, the *P*-values were then attributed to the *i*th gene using a ***χ*** squared distribution; *P*-values are 
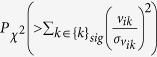
, where 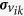 is the standard deviation of {*v*_*ik*_|*i* = 1, …, *N*}, and 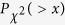 is the probability that the argument is larger than *x* under the assumption that the arguments obey a *χ* squared distribution. The *P*-values were further adjusted by the BH criterion^61^, and those genes associated with adjusted *P*-values less than 0.01 were selected as the genes associated with the difference between multiple classes. All the genes identified with PCA-based unsupervised FE are shown in S1_File.

### Gene expression profiles

Four *in vivo* gene expression data sets were downloaded from the Gene Expression Omnibus (GEO) using GEO IDs: GSE51808^33^, GSE13052^34^, GSE25001^35^, and GSE43777-GPL570/201^39^. Hereafter, these gene expression data will be denoted data sets 1, 2, 3, 4, and 5, respectively. One *in vitro* gene expression data set was also downloaded from GEO ID GSE9378^36^. The processed data GSEXXXXX_series_matrix.txt (where GSEXXXXX is GEO ID) for all five sets were downloaded and used for further analysis. Gene expression was scaled for PCA-based unsupervised FE, i.e., 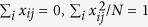. For other analyses, gene expression was used as it was, because the data had been processed. For details of the samples included in these gene expression profiles, see Table S1 in S1_Text.

### Biplot

A biplot is a scatter plot in which PC scores 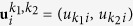
s and PC loadings 
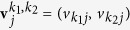
 are projected and over drawn onto the two-dimensional space spanned by the *k*_1_th and *k*_2_th PCs. For visibility (in other words, to avoid any overlap of the genes and samples or to avoid the locations of the genes or samples that are too close to the origin because the distances between the PC scores and PC loadings differ), an arbitrary positive constant scaling factor *c* is often used to multiply either **u**_*i*_s or **v**_*j*_s. By definition, since 
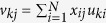
, the *j*th samples and *i*th genes that are oriented in the same direction from the origin are regarded as related on the biplot.

## Additional Information

**How to cite this article:** Taguchi, Y-H. Principal Components Analysis Based Unsupervised Feature Extraction Applied to Gene Expression Analysis of Blood from Dengue Haemorrhagic Fever Patients. *Sci. Rep.* **7**, 44016; doi: 10.1038/srep44016 (2017).

**Publisher's note:** Springer Nature remains neutral with regard to jurisdictional claims in published maps and institutional affiliations.

## Supplementary Material

Supplementary Text

Supplementary File

## Figures and Tables

**Table 1:** Forty-six genes identified with PCA-based unsupervised FE.

FBXO7	MX1	LY6E	IFI27	TNFSF10
OAS1	CDC20	GYPC	PI3	FCGR3A
HBA1	HBA2	HBG1	HBG2	IFI44L
IFIT3	CCR1	FPR1	STAT2	ISG15
OASL	CD38	TNFRSF17	CXCR1	ZBP1
HBB	IFI35	MKRN1	APOBEC3A	ALAS2
IL1RN	RSAD2	ASCC2	IFIT2	ADIPOR1
SLC25A37	OAS3	SDF2L1	TMEM140	FKBP11
HERC5	ITM2C	TXNDC5	STRADB	SLC25A39
EPSTI1				

**Table 2:** P-values that distinct DSS and uncomplicated patients in data set 4.

ACUTE		[0–1]	DIS	FOLLOWUP
PC2	2.14 × 10^−1^	5.62 × 10^−1^	7.87 × 10^−3^	4.15 × 10^−3^
PC3	7.23 × 10^−1^	1.07 × 10^−1^	6.41 × 10^−3^	9.73 × 10^−3^

*P*-values computed with a two-sample *t* test applied to the differences in the second and the third PC scores between “DSS” and “uncomplicated” patients. (Fig. 5).

**Table 3:** Enriched histone modification detected with Enrichr (ENCODE Histone Modifications 2015) in the 46 selected genes.

Rank	Histone modification	*P*-value	adjusted *P*-values	Z-score	combines score
1	H3K4me1_fibroblast of dermis_hg19	1.60E-06	6.20E-04	−1.8	13.29
2	H3K4me1_bone marrow macrophage_mm9	4.90E-05	3.16E-03	−1.87	10.74
3	H3K4me1_myotube_hg19	4.90E-05	3.16E-03	−1.7	9.78
4	**H3K4me1_K562_hg19**	2.56E-05	3.16E-03	−1.66	9.54
5	H3K4me1_A549_hg19	3.03E-05	3.16E-03	−1.64	9.43
6	H3K4me1_skeletal muscle myoblast_hg19	4.90E-05	3.16E-03	−1.62	9.33
7	**H3K4me2_K562_hg19**	2.33E-04	1.00E-02	−1.74	8.03
8	H3K4me1_HepG2_hg19	2.33E-04	1.00E-02	−1.68	7.75
9	H3K4me1_HeLa-S3_hg19	2.33E-04	1.00E-02	−1.6	7.37
10	H3K4me1_fibroblast of lung_hg19	9.88E-04	2.94E-02	−1.73	6.12
11	H3K4me2_HeLa-S3_hg19	9.88E-04	2.94E-02	−1.71	6.02
12	H3K4me1_T-cell acute lymphoblastic leukaemia_hg19	9.88E-04	2.94E-02	−1.64	5.8
13	H3K27ac_osteoblast_hg19	9.88E-04	2.94E-02	−1.63	5.75
14	H3K4me3_Panc1_hg19	1.40E-03	3.61E-02	−1.59	5.27
15	H3K4me1_MEL cell line_mm9	1.39E-03	3.61E-02	−1.47	4.88
16	H3K27ac_A549_hg19	1.95E-03	4.71E-02	−1.53	4.68
17	H3K4me3_A549_hg19	2.14E-03	4.88E-02	−1.51	4.56

Only those associated with adjusted *P*-values less than 0.05 are listed. Bold text indicates enrichment in the K562 cell line and is used in Fig. 1.
