# Supplementary material for: Principal Components Analysis Based Unsupervised Feature Extraction Applied to Gene Expression Analysis of Blood from Dengue Haemorrhagic Fever Patients
Source: Sci Rep. 2017 Mar 9;7:44016. doi: 10.1038/srep44016 (PMC5343617; doi:10.1038/srep44016)
Supplement: Supplementary Text [file srep44016-s1.pdf]

Principal Components Analysis Based on Unsupervised Feature Extraction Applied to Gene Expression Analysis of Blood from Dengue Haemorrhagic Fever Patients

Y-h. Taguchi[1,*]

[1]Department of Physics, Chuo University, Tokyo, 112-8551, Japan
[*]tag@granular.com

ABSTRACT

1 Theoretical background of PCA based unsupervised FE

Although it was empirically established that PCA based unsupervised FE worked well for a wide range of FEs/FSs when applied to gene expression/epigenetic profiles [1–14], the lack of theoretical background or justification has prevented other researchers from employing this methodology widely. It also prevented us from estimating in which circumstances it works well *a priori* (i.e., before applying this methodology to the specific problem). Here, we propose the theoretical background of this methodology for the first time based upon Ref. [15],which proved the equivalence between PCA and K-means, although Ding and He[15] did not recognize that their theoretical framework can be applicable to FS, because they applied PCA only to embedded samples, not to embedded features. In their paper, they proposed the K non-negative indicator vector $H_K = (\mathbf{h}_1, \cdots, \mathbf{h}_K)$, where

$$\mathbf{h}_k = (0, \cdots, 0, \overbrace{1, \cdots, 1}^{n_k}, 0, \cdots, 0)^T / n_k^{1/2}$$

and n_k is the size of the kth cluster, the elements with $h_k = 1$ belong to the kth cluster. They also showed that the connectivity matrix C is defined and represented as

$$C = \sum_{k=1}^{K} \mathbf{h}_k \mathbf{h}_k^T = \frac{1}{N} \mathbf{e}\mathbf{e}^T + \sum_{k=1}^{K-1} \mathbf{u}_k \mathbf{u}_k^T,$$

where $\mathbf{e} = (1, \cdots, 1)^T$. Thus, using PC scores, we could derive cluster structures among genes. In other words, embedding features by PCA is equivalent to figuring out how genes are clustered in the fully unsupervised manner. By computing C of synthetic data ($s = 2$) with $K = 1$, we could identify that the C between genes $i = 991, \cdots, 1000$, i.e., those with gene expression distinct between the two classes, have larger C values (see Fig. S6). This suggested that the theory proposed by Ref.[15] could be applicable not only to sample classification, as they have done, but also to FSs, as has been demonstrated in PCA based unsupervised FE. Thus, Ding and He[15] provided the theoretical background as to why PCA based unsupervised FE works well; PCA based unsupervised FE tries to cluster genes that share similar expression profiles and this results in the selection of genes with expressions distinct between two classes. One may wonder why we do not use directly K-means instead of PCA, if they are equivalent. The reason is that there is only one small cluster to which a limited number of (in this case, as small as 10) genes belong, while the majority (99 % genes) do not form any clusters. K-means must cluster all genes without exceptions. This forces K-means to generate non-existent clusters. Actually, if we apply K-means assuming two clusters, we could never obtain a small cluster including only 10 genes, instead we have two broad clusters whose sizes are equivalent to each other. This kind of methodological limitation exists in all clustering methodologies, because all clustering methodology must cluster elements into a limited number of clusters, even when there is only one small cluster to which a very small part of the elements belong and majority do not form any clusters at all. To the best of our knowledge, PCA based unsupervised FE is the only methodology that could deal with this kind of difficult-to-treat situation. This is possibly the reason why PCA based unsupervised FE could outperform other conventional methodologies for a wide range of problems. In addition, Ding and He[15] provided the missing criteria concerning in which circumstances PCA based unsupervised FE is recommended. Simply speaking, if there is a limited number of small clusters to which a limited proportion of elements belong while the majority of elements do not form any

clusters, PCA based unsupervised FE is useful. To see if this is the case for a DENV data set, we computed C for data set 2 with $K = 3$ and ordered columns/rows using the spectral ordering[16] (Fig. S6). The results definitely showed that situation is even worse; there are no clear clusters (block diagonal parts) although around the corners there are some genes with high connectivities, because if there are clusters, multiple block diagonal structures should appear as demonstrated by Refs.[15,16]. This is possibly why PCA based unsupervised FE could outperform those methodologies that cannot deal with this situation effectively. It also supports the employment of two minor PCs (2nd and 3rd) that result in the clear appearance of a set of genes associated with high connectivity. Furthermore, we computed the correlation coefficient between $\mathbf{q}_1$, that is the *continuous* inverse index permutation[16] and is used to order features, and P-values attributed to each gene by PCA based unsupervised FE. Correspondingly, high correlation (Pearson's correlation coefficient is as large as 0.627, that is associated with $P < 2.2 \times 10^{-16}$) was observed. This supported the use of P-values for FS instead of $\mathbf{q}_1$, which requires diagonalization of an $N \times N$ (thus, generally huge) matrix. We believe the discussion in this subsection justifies the use of PCA based unsupervised FE for the difficult situation where there are only a few (or even no) clusters to which a limited number of elements belong while the majority of elements do not form any clusters, which was a difficult situation that could not be dealt with well by other methods. For more details about the computation of C, see below.

2 Additional methodological advantages of PCA based unsupervised FE

In the previous subsection, we discussed the general methodological advantages of PCA based unsupervised FE based upon the theory proposed by[15]. There are several additional advantages of PCA based unsupervised FE. For example, one may wonder why genes were not screened directly based on the criteria used to specify the PCs for FEs, i.e., DHF+DF vs. CP+HC or convalescent vs. acute. Other than the problem that there are too many genes identified (see above), selecting genes because of their fitness to assumed categorical classes is problematic. It was impossible to reconstruct a two-dimensional space where DHF and DF were well discriminated, as has been done in the present research. Fig. S7 shows the distribution of genes attributed to the two classes on the plane spanned by the PC2 and PC3 loadings. It is obvious that the genes are not unidirectionally distributed around the origin, but alongside the diagonal directions; this is the direction that mostly represents the distinction between the two classes. This means that to construct a two-dimensional space where DF and DHF were well discriminated, we need to include genes unrelated to the distinction between the two classes. This shows the limitation of the supervised method, which can select something targeted, while unsupervised FE can depict something not intended but related to the critical biological background. No supervised method can overcome this difficulty because they cannot select genes that are not specific to something targeted. It is unrealistic to assume that we know everything; therefore, a supervised method might miss something biologically important unintentionally. Thus, unsupervised methods are preferable to supervised if the unsupervised method can be applied to the data set.

Another advantage of the unsupervised method is the number of classes that should be assumed when FE is performed. Although data sets 1 and 2 apparently comprise four classes, in our analysis, we identified that two classes is a reasonable assumption. However, it is difficult for supervised methods to assume a suitable number of classes, because the number of classes is not supposed to be identified, but to be assumed by supervised methodology. Thus, it is evident that assuming two classes not four classes in data sets 1 and 2 is the reason of the successful FEs, and unsupervised FE is more suitable to the present study than supervised FEs.

Furthermore, although PCA is supposed not to be able to represent non-linearity, because PCA is a linear method, this is not always true. For example, in Fig. 4 in main text, development time of diseases is not proportional to any of the gene expressions, because it curves. However, since PCA identifies a two-dimensional space where non-linearity can be expressed as a curve, PCA identified successfully the non-linear dependence of development time upon gene expression. In this sense, if PCA could detect more than one-dimensional space, e.g., a plane, non-linearity could be captured, even using linear methods like PCA.

3 Details about sam and limma

When using sam, gene expression is given to `sam` assuming two or four class arrangements. Then probes associated with `q.value` less than 0.01 were identified as selected genes. Wehn using limma assuming two classes, pseudo R code is

```
gene_exp <- new("ExpressionSet",expr=data.matrix(log(x[,-1])))
fData(gene_exp)[["gene_id"]] <- x[,1]
pData(gene_exp)[["sample_name"]]<- class
design <- model.matrix(~0+class)
colnames(design) <- levels(class)
fit <- lmFit(gene_exp, design)
fit <- eBayes(fit)
```

```
#------ four two classes ------
TT <- topTableF(fit, adjust="BH",number=dim(x)[1])
#------- for four classes ----
contrast.matrix <- makeContrasts(class1-class2,class1-class3,class1-class4,class2-class3,
 class2-class4,class3-class4, levels=design)
fit2 <- contrasts.fit(fit, contrast.matrix)
fit2 <- eBayes(fit2)
TT <- topTableF(fit2, adjust="BH",number=dim(x)[1])
#----------------------
table(TT[,6]<0.01)
```

where x is supposed to include gene expression, with rows and columns being genes and samples, respectively. The first column is supposed to be gene identifier. `class` is supposed to be factor that represents sample classes. `TT[,6]` is supposed to include adjusted P-values.

4 Details of computing connectivity matrix C

4.1 Synthetic data set

For synthetic data set, a 1000×1000 matrix was generated as described in main text. Then, C is computed. Fig. S6 shows the connectivity matrix between 900th and 1000th genes. Only genes between 990th and 1000th are associated with distinct expression between two classes.

4.2 Data set 2

After computing connectivity matrix C, the eigen vector $\mathbf{q}_1$ was computed. Starting initial random vector $\mathbf{q}_1$ drawn from the uniform distribution $(0,1]$, only three iterations of $\mathbf{q}_1 \leftarrow C\mathbf{q}_1$ with suitable scaling $|\ \mathbf{q}_1\ |= 1$ turned out to be enough for the convergence. Since $-\mathbf{q}_1$ is also an eigen vector if $\mathbf{q}_1$ is an eigne vector, we could not identify if gene are ordered in the decreasing or increasing order of the elements of $\mathbf{q}_1$, we first compute the correlation between $\mathbf{q}_1$ and P-vales that PCA based unsupervised FE attributed to each gene. Then, genes are ordered such that those associated with smaller P-values are top ranked. Then, connectivities among top ranked 2400 genes are drawn in Fig. S6 after averaging over every 10 sequentially ranked genes.

References

1. Taguchi, Y.-h. Identification of aberrant gene expression associated with aberrant promoter methylation in primordial germ cells between E13 and E16 rat F3 generation vinclozolin lineage. *BMC Bioinformatics* **16**, S16 (2015). URL http://www.biomedcentral.com/1471-2105/16/S18/S16.
2. Taguchi, Y.-h. Integrative analysis of gene expression and promoter methylation during reprogramming of a non-small-cell lung cancer cell line using principal component analysis-based unsupervised feature extraction. In Huang, D.-S., Han, K. & Gromiha, M. (eds.) *Intelligent Computing in Bioinformatics*, vol. 8590 of *LNCS*, 445–455 (Springer International Publishing, Heidelberg, 2014).
3. Taguchi, Y.-h., Iwadate, M., Umeyama, H., Murakami, Y. & Okamoto, A. Heuristic principal component analysis-aased unsupervised feature extraction and its application to bioinformatics. In Wang, B., Li, R. & Perrizo, W. (eds.) *Big Data Analytics in Bioinformatics and Healthcare*, 138–162 (2015).
4. Taguchi, Y.-H., Iwadate, M. & Umeyama, H. Heuristic principal component analysis-based unsupervised feature extraction and its application to gene expression analysis of amyotrophic lateral sclerosis data sets. In *Computational Intelligence in Bioinformatics and Computational Biology (CIBCB), 2015 IEEE Conference on*, 1–10 (2015).
5. Taguchi, Y. H., Iwadate, M. & Umeyama, H. Principal component analysis-based unsupervised feature extraction applied to in silico drug discovery for posttraumatic stress disorder-mediated heart disease. *BMC Bioinformatics* **16**, 139 (2015).
6. Umeyama, H., Iwadate, M. & Taguchi, Y. H. TINAGL1 and B3GALNT1 are potential therapy target genes to suppress metastasis in non-small cell lung cancer. *BMC Genomics* **15 Suppl 9**, S2 (2014).
7. Murakami, Y. *et al.* Comprehensive analysis of transcriptome and metabolome analysis in Intrahepatic Cholangiocarcinoma and Hepatocellular Carcinoma. *Sci Rep* **5**, 16294 (2015).
8. Murakami, Y. *et al.* Comparison of Hepatocellular Carcinoma miRNA Expression Profiling as Evaluated by Next Generation Sequencing and Microarray. *PLoS ONE* **9**, e106314 (2014).

9. Murakami, Y. *et al.* Comprehensive miRNA expression analysis in peripheral blood can diagnose liver disease. *PLoS ONE* **7**, e48366 (2012).

10. Taguchi, Y. H. & Murakami, Y. Universal disease biomarker: can a fixed set of blood microRNAs diagnose multiple diseases? *BMC Res Notes* **7**, 581 (2014).

11. Taguchi, Y. H. & Murakami, Y. Principal component analysis based feature extraction approach to identify circulating microRNA biomarkers. *PLoS ONE* **8**, e66714 (2013).

12. Kinoshita, R., Iwadate, M., Umeyama, H. & Taguchi, Y. H. Genes associated with genotype-specific DNA methylation in squamous cell carcinoma as candidate drug targets. *BMC Syst Biol* **8 Suppl 1**, S4 (2014).

13. Ishida, S., Umeyama, H., Iwadate, M. & Taguchi, Y. H. Bioinformatic Screening of Autoimmune Disease Genes and Protein Structure Prediction with FAMS for Drug Discovery. *Protein Pept. Lett.* **21**, 828–39 (2014).

14. Taguchi, Y.-h. & Okamoto, A. Principal component analysis for bacterial proteomic analysis. In Shibuya, T., Kashima, H., Sese, J. & Ahmad, S. (eds.) *Pattern Recognition in Bioinformatics*, vol. 7632 of *LNCS*, 141–152 (Springer International Publishing, Heidelberg, 2012).

15. Ding, C. & He, X. K-means clustering via principal component analysis. In *Proceedings of the Twenty-first International Conference on Machine Learning*, ICML '04, 29– (ACM, New York, NY, USA, 2004). URL http://doi.acm.org/10.1145/1015330.1015408.

16. Ding, C. & He, X. Linearized cluster assignment via spectral ordering. In *Proceedings of the Twenty-first International Conference on Machine Learning*, ICML '04, 30– (ACM, New York, NY, USA, 2004). URL http://doi.acm.org/10.1145/1015330.1015407. DOI:10.1145/1015330.1015407.

Table S1. List of samples included in data set 1, 2 and 3. DSS:Dengue Shock Syndrome. GSE51808: RMA normalization was performed using Expression Console software. GSE13052: Intensity was acquired using Beadstudio software Intensity was background normalised (Subtract the background value). GSE25001: Data was normalised by Beadstudio software. GSE9378: Signal values were calculated using robust multi-array analysis (RMA) (BioConductor), transformed using inverse nlog, and then imported into GeneSpring (Agilent) for chip normlaization to 50th percentile and gene normalization to the mean of controls (where available) for each cell type independently. GSE43777: RMA normalization was performed using Expression Console software. For more details, see paper.

Data set 1 (GSE51808)	Affymetrix HT HG-U133+ PM Array Plate			
	Healthy Controls (HC)	Acute Patients (AC)	DF	DHF
	9	19	18	10
Data set 2 (GSE13052)	Sentrix HumanRef-8 Expression BeadChip			
	Acute	Convalescent		
uncomplicated (DF)	10	5		
DSS* (DHF)	9	6		
Data set 3 (GSE25001)	Illumina humanRef-8 v2.0 expression beadchip			
	Acute	0-1	Disease (Fever)	follow up
DF	56	32	31	16
DHF	24	12	20	18
in vitro (GSE9378)	Affymetrix Human Genome U133A Array			
	HUVEC	Monocyte		
control	2	2		
infected	2	2		

Data set 4 (GSE43777-GPL570)	Affymetrix Human Genome U133 Plus 2.0 Array							
	G0	G1	G2	G3	G4	G5	G6	G7
DF	0	2	5	8	9	5	11	12
DHF	0	0	3	8	10	5	11	12
Data set 5 (GSE43777-GPL201)	Affymetrix Human HG-Focus Target Array							
DF	2	5	21	18	22	22	24	45
DHF	0	0	0	1	3	1	1	3

Table S2. Number of genes identified by sam, limma and PCA based unsupervised FE. Two classes mean "DHF+DF" vs "CP+HC" for data set 1 (GSE51808) and "Acute" vs "Convalescent" for data set 2 (GSE13052). *:all probes. The numbers in parentheses are those when the sample numbers are halved. Averaged values over 100 ensembles are presented. Halving was performed within each of four classes. Thus, the ratio between classes was conserved.

Data set	sam		limma		PCA based unsupervised FE
	two classes	four classes	two classes	four classes	
1	17680 (18469)	16647 (7461)	54715* (54715*)	13506 (5706)	879 (826)
2	2427 (41)	865 (0)	21795 (19855)	20629 (17478)	275 (286)

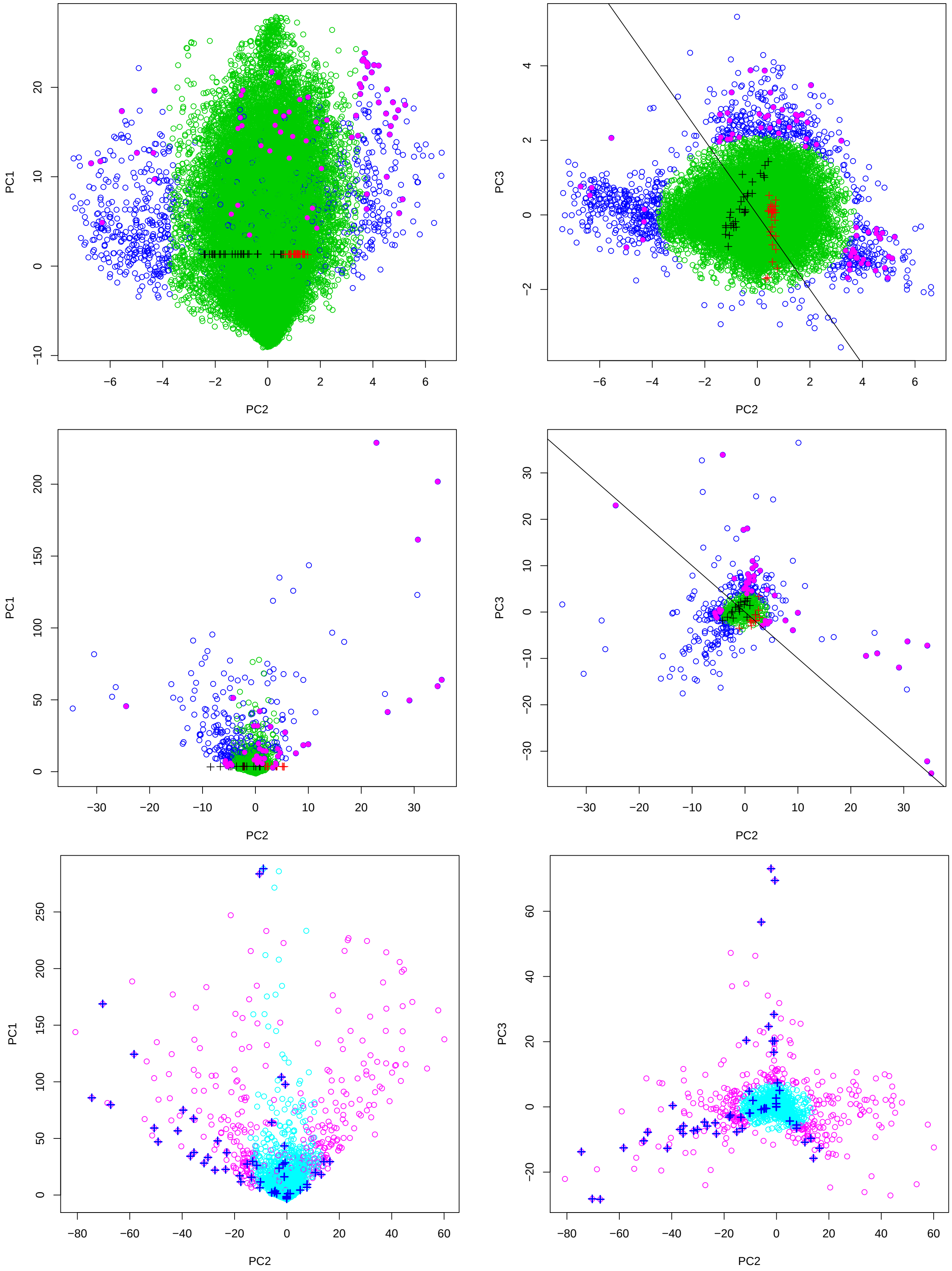

Figure S1. **Top**: Biplot of PC1 to PC3 for data set 1 (GSE51808). Open green circles are probes not selected as outliers. Open blue circles are 879 probes identified as outliers. Black and red crossed represent patients with symptom (DF/DHF) and those without symptom (HC/AC). Solid line represents the line PC2=-PC3 that roughly represents the distinction between patients with/without symptom. Open magenta circles are probes associated with 46 genes commonly identified as outliers in data set 1 and 2. **Middle**: Biplot of PC1 to PC3 for data set 2 (GSE13052). Open green circles are probes not selected as outliers. Open blue circles are 275 probes identified as outliers. Black and red crossed represent patients with symptom (acute) and those without symptom (convalescent). Solid line represents the line PC2=-PC3 that roughly represents the distinction between patients with/without symptom. Open magenta circles are probes associated with 46 genes commonly identified as outliers in data set 1 and 2. **Bottom**: Scatter plot of PC1 to PC3 scores attributed to probes for data set 3 (GSE25001). Open cyan circles are probes not selected as outliers. Open magenta circles are probes identified as outliers. Blue crossed represents probes associated with 46 genes commonly identified as outliers in data set 1 and 2.

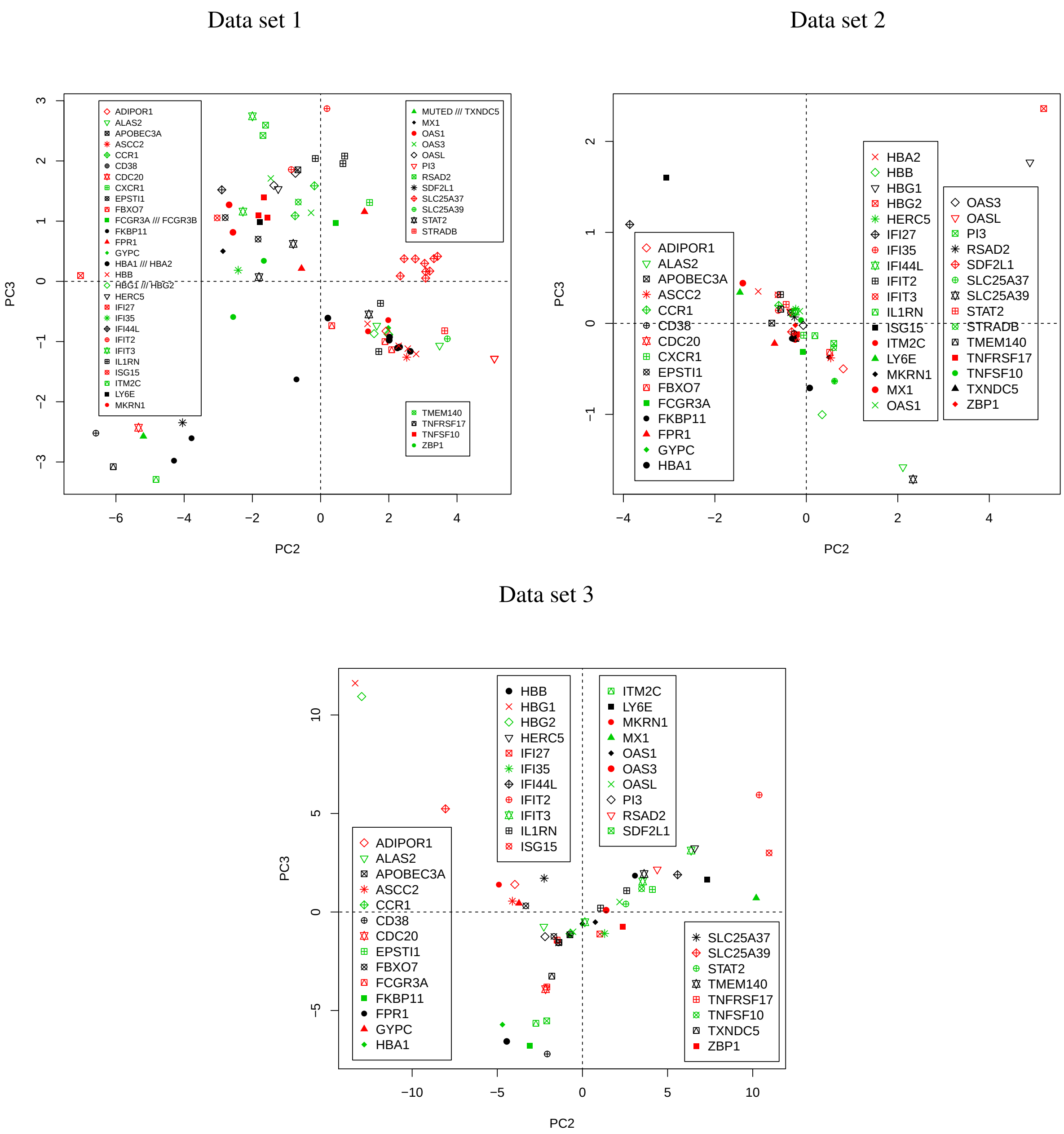

Figure S2. Annotation of genes shown in Figs. 4 (upper) and data set 3 in Fig. 5(lower).

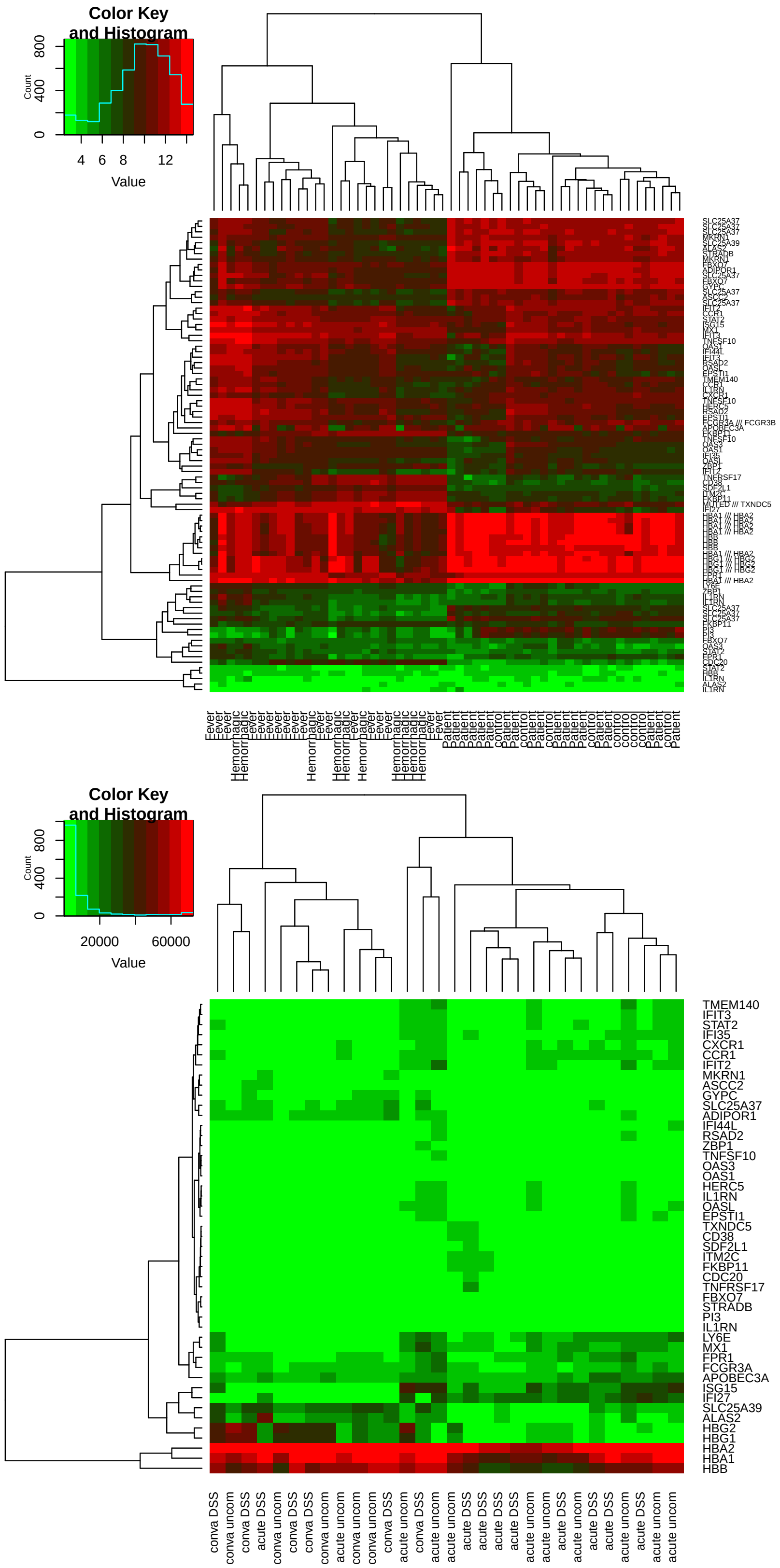

Figure S3. Heatmaps of probes associated with 46 genes commonly identified in data set 1 and 2. Upper: Data set 1. Samples are strictly grouped as "Fever" (DF) + "hemorrhagic" (DHF) and control (HC) + Patient (CP). Lower: Data set 2. Samples are almost grouped as "conva" (convalescent) and "acute". Only two acute and one conva patients were wrongly grouped. Grading: bright red (green) represents more expressive(suppressive) expressions. They are drawn using heatmap function implemented in R. hierarchical clustering was performed by Unweighted Pair Group Method with Arithmetic mean with setting method = "average" option. Distances between gene expression were Euclidean distance.

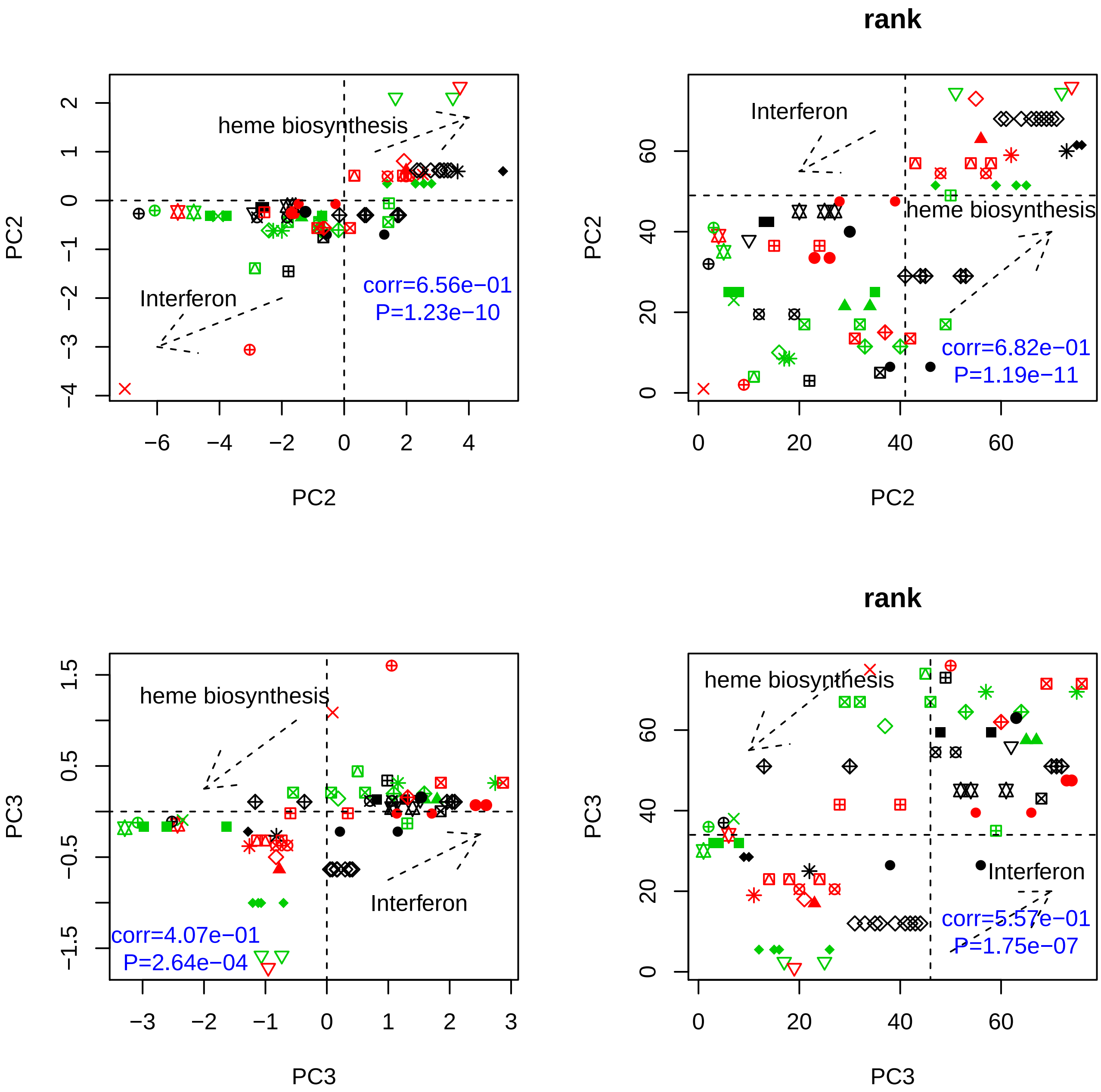

Figure S4. Comparison between PC scores attributed to probes associated with 46 genes commonly identified in data set 1 and 2. Upper:PC2, lower:PC3, Left:PC scores, right: rank of PC scores. Correlation coefficients (left:Pearson, right:Spearman), as well as associated *P*-values are also shown. For the annotation of characters, see Fig. S5

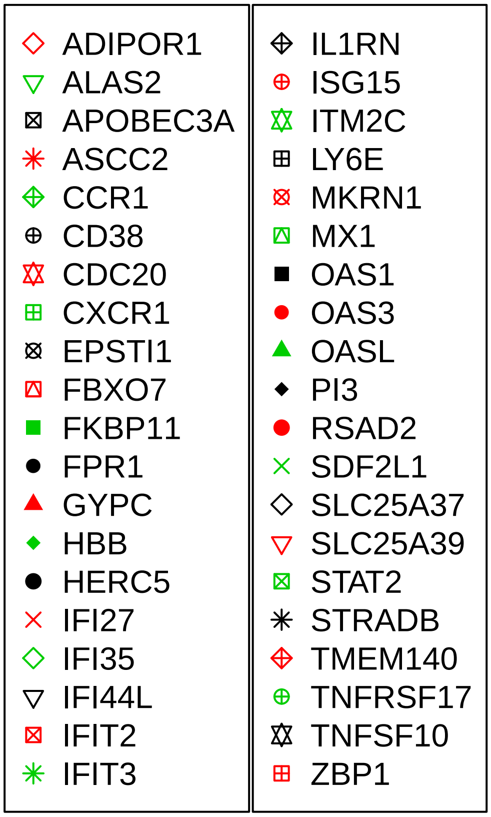

Figure S5. Character annotations used in Fig. S4

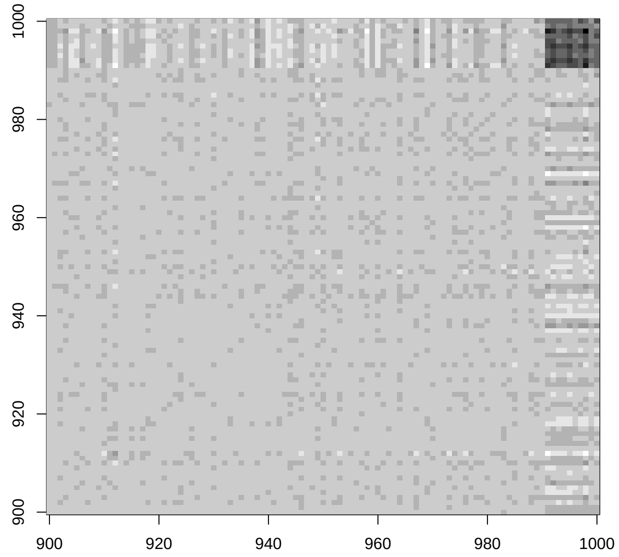

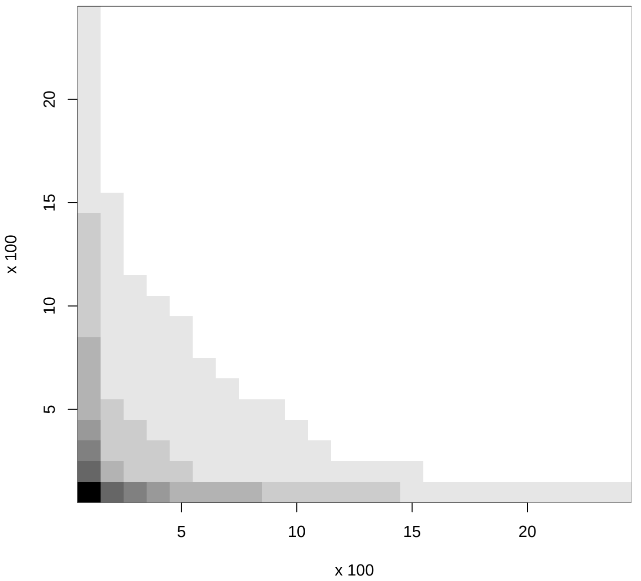

Figure S6. Connectivity matrix of synthetic data ($s = 2$, left, genes $900 \leq i \leq 1000$) and data set 2 (right, coarse grained values averaged over every 10 sequentially ranked genes among top ranked 2400 genes). Darker gray scales correspond to higher connectivities.

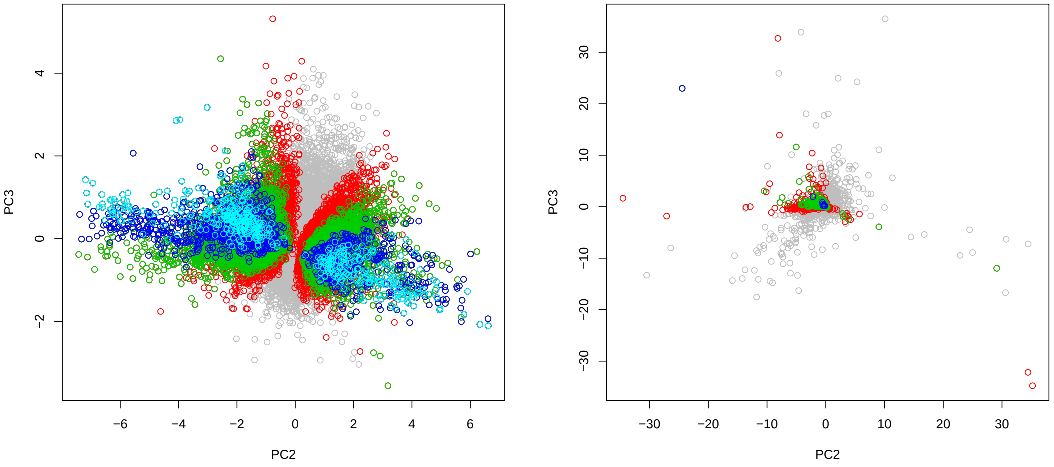

Figure S7. Left:Distribution of P-values adjusted by BH criterion on two dimensional space spanned by PC2 and PC3 loadings for data set 1 (GSE51808). P-values were computed by t test and were those to deny null hypothesis that mean of x_{ij} within DF+DHF are identical to that of of x_{ij} within CP+HC. Red open circles: 1×10^{-5} < adjusted P-values < 0.01, Green open circles: 1×10^{-10} < adjusted P-values < 1×10^{-5}, blue open circles: 1×10^{-13} < adjusted P-values < 1×10^{-10}, cyan open circles: adjusted P-values < 1×10^{-13}. Right: Distribution of same variables but for data set 2 (GSE13052). P-values were computed by t test and were those to deny null hypothesis that mean of x_{ij} within convalescent are identical to that of of x_{ij} within acute. Red open circles: 1×10^{-3} < adjusted P-values < 0.01, Green open circles: 1×10^{-4} < adjusted P-values < 1×10^{-3}, blue open circles: adjusted P-values < 1×10^{-4}.

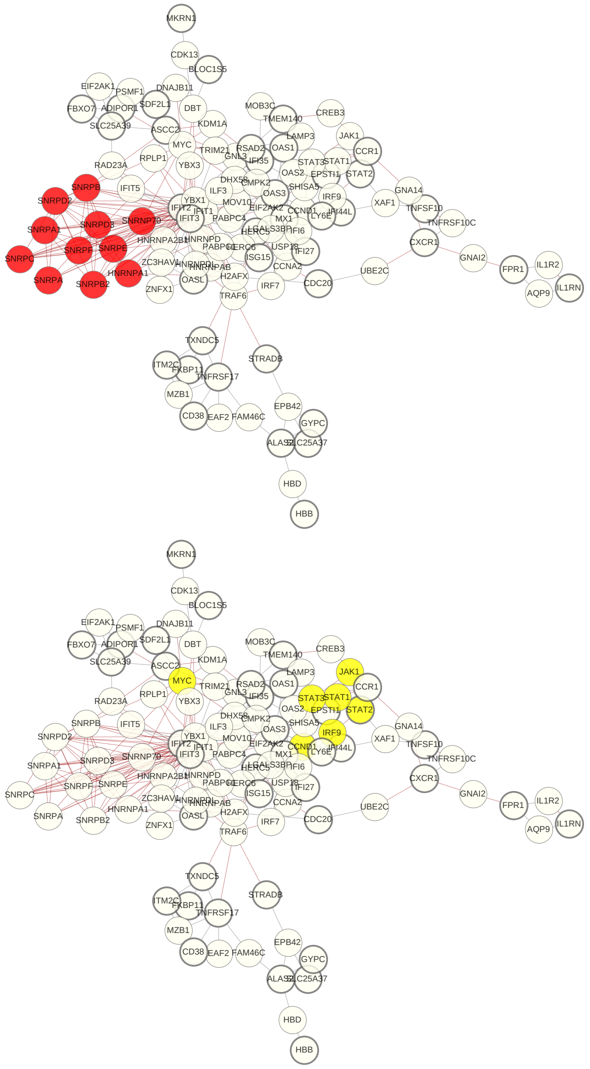

Figure S8. Co-expression network inferred by COEXPRESSdb. Upper(red):Spliceosome(hsa03040), lower(yelow):Jak-STAT signaling pathway(hsa04630). Genes in bold open circles are 46 genes identified by PCA based unsupervised FE

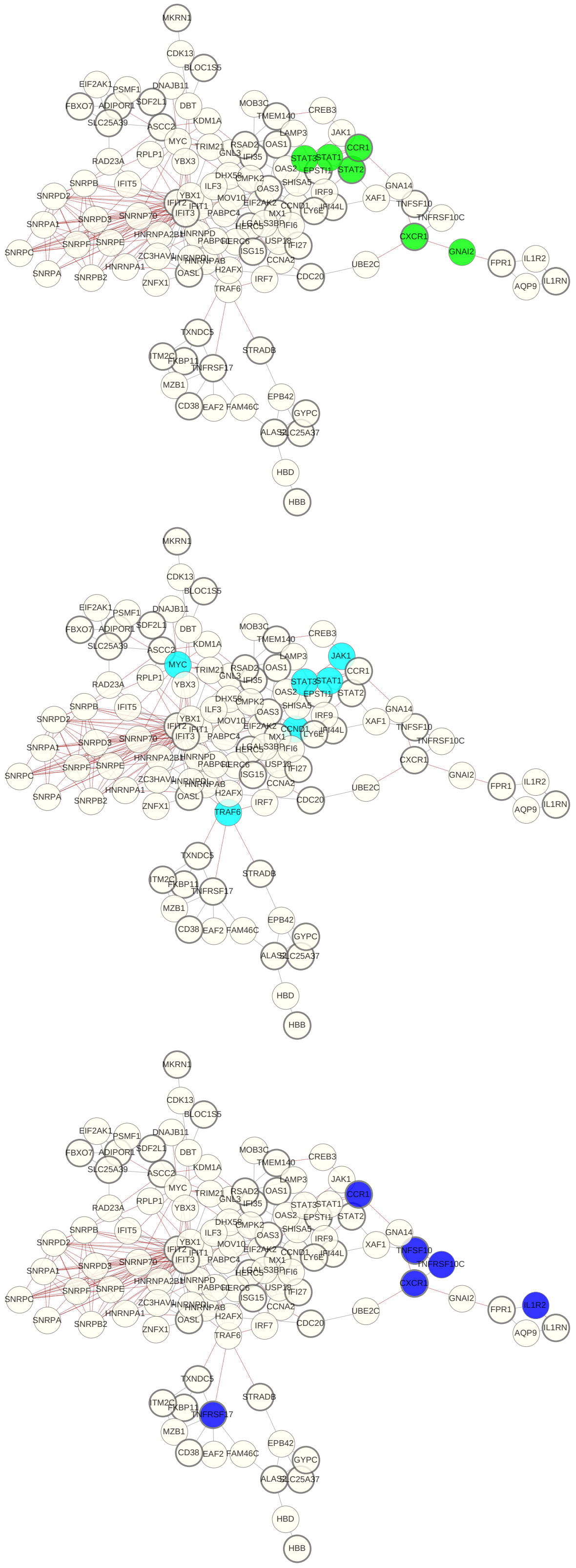

Figure S9. Co-expression network inferred by COEXPRESSdb (continued). Upper(green): Chemokine signaling pathway(hsa04062), middle(cyan): Pathways in cancer(hsa05200), lower(blue) Cytokine-cytokine receptor interaction (hsa04060). Genes in bold open circles are 46 genes identified by PCA based unsupervised FE

source	term name<br>Gene Ontology (Biological process)	term ID	n. of term genes	corrected p-value	OURS	LONG_ET_AL
BP	regulation of multi-organism process	GO:0043900	479	3.69e-03	9	
BP	regulation of symbiosis, encompassing mutualism through parasitism	GO:0043903	218	1.85e-03	7	
BP	regulation of viral process	GO:0050792	194	8.40e-04	7	
BP	regulation of viral life cycle	GO:1903900	181	5.25e-04	7	
BP	negative regulation of multi-organism process	GO:0043901	148	1.32e-04	7	
BP	viral genome replication	GO:0019079	90	4.09e-06	7	
BP	regulation of viral genome replication	GO:0045069	71	7.53e-07	7	
BP	negative regulation of viral process	GO:0048525	87	3.22e-06	7	
BP	negative regulation of viral life cycle	GO:1903901	84	2.51e-06	7	
BP	negative regulation of viral genome replication	GO:0045071	47	3.71e-08	7	
BP	cell division	GO:0051301	625	2.98e-07		18
BP	cell cycle	GO:0007049	1742	3.90e-09		31
BP	mitotic cell cycle	GO:0000278	1000	5.91e-09		24
BP	chromosome segregation	GO:0007059	269	3.03e-07		13
BP	regulation of chromosome segregation	GO:0051983	86	4.79e-03		6
BP	organelle organization	GO:0006996	3556	1.01e-02		33
BP	organelle fission	GO:0048285	556	3.28e-11		21
BP	nuclear division	GO:0000280	525	1.06e-11		21
BP	single-organism organelle organization	GO:1902589	2543	9.89e-03		27
BP	chromosome condensation	GO:0030261	31	2.36e-02		4
BP	negative regulation of organelle organization	GO:0010639	415	2.84e-02		10
BP	chromosome organization	GO:0051276	1077	2.89e-02		16
BP	cell cycle process	GO:0022402	1262	3.43e-10		28
BP	nuclear chromosome segregation	GO:0098813	207	3.36e-06		11
BP	sister chromatid segregation	GO:0000819	149	3.86e-05		9
BP	cell cycle checkpoint	GO:0000075	244	2.60e-03		9
BP	cell cycle phase transition	GO:0044770	494	2.10e-02		11
BP	mitotic cell cycle process	GO:1903047	831	8.87e-09		22
BP	mitotic cell cycle phase transition	GO:0044772	471	1.34e-02		11
BP	mitotic nuclear division	GO:0007067	409	3.38e-09		17
BP	mitotic sister chromatid segregation	GO:0000070	124	1.53e-04		8
BP	regulation of cell cycle	GO:0051726	1006	8.98e-05		19
BP	regulation of cell cycle process	GO:0010564	524	8.58e-04		13
BP	microtubule cytoskeleton organization	GO:0000226	404	2.25e-02		10
BP	microtubule cytoskeleton organization involved in mitosis	GO:1902850	45	3.55e-03		5
BP	microtubule polymerization or depolymerization	GO:0031109	188	3.72e-03		8
BP	microtubule depolymerization	GO:0007019	153	1.09e-02		7
BP	regulation of microtubule polymerization or depolymerization	GO:0031110	171	2.26e-02		7
BP	negative regulation of microtubule polymerization or depolymerization	GO:0031111	151	9.97e-03		7
BP	spindle organization	GO:0007051	123	3.82e-02		6
BP	mitotic spindle assembly	GO:0090307	45	3.55e-03		5
BP	biological_process	GO:0008150	16636	2.25e-02		82
BP	immune system process	GO:0002376	2587	4.80e-06	23	29
BP	immune effector process	GO:0002252	759	3.15e-05	13	13
BP	response to stimulus	GO:0050896	8357	1.67e-03	36	
BP	response to chemical	GO:0042221	4374	2.19e-02	24	
BP	cellular response to chemical stimulus	GO:0070887	2852	2.30e-02	19	
BP	response to organic substance	GO:0010033	3004	4.93e-02	19	
BP	response to cytokine	GO:0034097	847	7.18e-09	17	14
BP	response to interferon-alpha	GO:0035455	19	3.00e-02	3	
BP	cellular response to organic substance	GO:0071310	2368	6.89e-03	18	
BP	cellular response to cytokine stimulus	GO:0071345	747	1.42e-08	16	
BP	cellular response to interferon-alpha	GO:0035457	10	3.77e-03	3	
BP	response to stress	GO:0006950	3974	6.83e-07	29	38
BP	defense response	GO:0006952	1800	1.12e-04	18	
BP	response to biotic stimulus	GO:0009607	943	3.14e-03	12	
BP	immune response	GO:0006955	1679	5.05e-06	19	21
BP	innate immune response	GO:0045087	1097	3.06e-04	14	
BP	response to interferon-gamma	GO:0034341	154	1.14e-02		7
BP	response to type I interferon	GO:0034340	81	7.59e-12	10	
BP	cellular response to type I interferon	GO:0071357	79	5.84e-12	10	
BP	response to external biotic stimulus	GO:0043207	905	2.03e-03	12	
BP	response to other organism	GO:0051707	905	2.03e-03	12	
BP	response to virus	GO:0009615	407	2.86e-07	12	
BP	defense response to other organism	GO:0098542	553	9.35e-06	12	
BP	defense response to virus	GO:0051607	325	2.11e-08	12	
BP	cell surface receptor signaling pathway	GO:0007166	2758	1.03e-04	22	
BP	cytokine-mediated signaling pathway	GO:0019221	604	5.63e-10	16	12
BP	type I interferon signaling pathway	GO:0060337	79	5.84e-12	10	
BP	gas transport	GO:0015669	20	2.25e-06	5	
BP	oxygen transport	GO:0015671	15	4.40e-07	5	

source	term name<br>Gene Ontology (Cellular component)	term ID	n. of term genes	corrected p-value	OURS	LONG_ET_AL
CC	chromosome	GO:0005694	860	1.59e-03		16
CC	condensed chromosome	GO:0000793	193	3.60e-04		9
CC	endocytic vesicle lumen	GO:0071682	17	2.11e-02	3	
CC	nuclear lumen	GO:0031981	3405	3.55e-02		31
CC	chromosomal region	GO:0098687	303	1.53e-02		9
CC	chromosome, centromeric region	GO:0000775	172	1.90e-03		8
CC	cytosol	GO:0005829	3200	7.17e-03	21	31
CC	chromosome passenger complex	GO:0032133	5	1.97e-03		3
CC	hemoglobin complex	GO:0005833	12	1.17e-07	5	3
CC	haptoglobin-hemoglobin complex	GO:0031838	4	1.27e-04	3	

source	term name<br>Gene Ontology (Molecular function)	term ID	n. of term genes	corrected p-value	OURS	LONG_ET_AL
MF	2'-5'-oligoadenylate synthetase activity	GO:0001730	3	4.25e-02	2	
MF	tetrapyrrole binding	GO:0046906	136	4.27e-02	5	
MF	heme binding	GO:0020037	127	3.06e-02	5	
MF	oxygen transporter activity	GO:0005344	14	2.94e-07	5	
MF	oxygen binding	GO:0019825	38	7.06e-05	5	
MF	haptoglobin binding	GO:0031720	3	3.18e-05	3	

source	term name<br>Protein databases (CORUM protein complexes)	term ID	n. of term genes	corrected p-value	OURS	LONG_ET_AL
cor	Chromosomal passenger complex CPC (CDCA8, AURKB, BIRC5)	CORUM:2582	3	1.27e-04		3
cor	CRM1-Survivin-AuroraB mitotic complex	CORUM:1116	3	5.00e-02		2
cor	Chromosomal passenger complex CPC (INCENP, CDCA8, BIRC5, AURKB)	CORUM:1118	4	5.03e-04		3
cor	Chromosomal passenger complex CPC (INCENP, CDCA8, BIRC5, AURKB)	CORUM:1119	4	5.03e-04		3
cor	Chromosomal passenger complex CPC (INCENP, BIRC5, AURKB)	CORUM:2579	3	5.00e-02		2
cor	Chromosomal passenger complex CPC (INCENP, CDCA8, BIRC5)	CORUM:1120	3	5.00e-02		2

source	term name<br>Human Phenotype Ontology	term ID	n. of term genes	corrected p-value	OURS	LONG_ET_AL
hp	Cyanosis	HP:0000961	65	2.96e-02	4	
hp	Polycythemia	HP:0001901	20	2.36e-02	3	
hp	Abnormal hemoglobin	HP:0011902	29	1.10e-05	5	
hp	Hemoglobin H	HP:0011903	3	2.50e-02	2	
hp	Persistence of hemoglobin F	HP:0011904	22	3.17e-02	3	
hp	Imbalanced hemoglobin synthesis	HP:0005560	6	4.23e-04	3	
hp	Reduced alpha/beta synthesis ratio	HP:0011907	3	2.50e-02	2	
hp	Methemoglobinemia	HP:0012119	5	2.12e-04	3	
hp	Anemia due to reduced life span of red cells	HP:0011895	115	1.26e-02	5	
hp	Hemolytic anemia	HP:0001878	115	1.26e-02	5	
hp	Nonspherocytic hemolytic anemia	HP:0001930	7	7.39e-04	3	
hp	Heinz body anemia	HP:0005511	3	2.13e-05	3	
hp	Pallor	HP:0000980	150	4.60e-02	5	
hp	Decreased serum complement C4	HP:0045042	9	2.51e-02		3
hp	Decreased serum complement C4b	HP:0045044	9	2.51e-02		3
hp	Chronic active hepatitis	HP:0200120	11	4.89e-02		3
hp	Hashimoto thyroiditis	HP:0000872	11	4.89e-02		3
hp	Anemia of inadequate production	HP:0010972	94	1.19e-04	6	
hp	Microcytic anemia	HP:0001935	29	7.54e-08	6	
hp	Hypochromic anemia	HP:0001931	23	4.15e-04	4	
hp	Hypochromic microcytic anemia	HP:0004840	8	3.36e-06	4	
hp	Abnormality of the heme biosynthetic pathway	HP:0010472	23	1.61e-08	6	
hp	Triangular nasal tip	HP:0000451	3	2.50e-02	2	
hp	Hypersplenism	HP:0001971	11	3.46e-03	3	

source	term name<br>Protein databases (Human Protein Atlas)	term ID	n. of term genes	corrected p-value	OURS	LONG_ET_AL
hpa	soft tissue 2; peripheral nerve	HPA:040040	6967	2.27e-02		52

source	term name<br>Biological pathways (KEGG)	term ID	n. of term genes	corrected p-value	OURS	LONG_ET_AL
keg	African trypanosomiasis	KEGG:05143	34	9.71e-03	3	
keg	Measles	KEGG:05162	136	3.73e-03	5	
keg	Influenza A	KEGG:05164	174	8.88e-04	6	
keg	Malaria	KEGG:05144	48	8.87e-04	4	

source	term name<br>Regulatory motifs in DNA (miRBase microRNAs)	term ID	n. of term genes	corrected p-value	OURS	LONG_ET_AL
mi	MI:hsa-miR-922	MI:hsa-miR-922	633	3.68e-02		11

source	term name<br>Online Mendelian Inheritance in Man	term ID	n. of term genes	corrected p-value	OURS	LONG_ET_AL
omi	HEINZ BODY ANEMIAS	OMIM:140700	3	2.01e-06	3	
omi	Hydrops Fetalis	OMIM:236750	9	2.00e-02	2	
omi	HEMOGLOBIN H DISEASE; HBH;;ALPHA-THALASSEMIA, HEMOGLOBIN H TYPE;;HEMOG...	OMIM:613978	2	5.65e-04	2	
omi	FETAL HEMOGLOBIN QUANTITATIVE TRAIT LOCUS 1; HBFQTL1;;HEMOGLOBIN F, HERE ...	OMIM:141749	3	2.01e-06	3	2
omi	ALPHA-THALASSEMIA	OMIM:604131	2	5.65e-04	2	

source	term name<br>Biological pathways (Reactome)	term ID	n. of term genes	corrected p-value	OURS	LONG_ET_AL
rea	SUMOylation of DNA replication proteins	REAC:4615885	45	4.66e-02		4
rea	Immune System	REAC:168256	1566	2.84e-04	18	
rea	Cytokine Signaling in Immune system	REAC:1280215	625	2.61e-06	14	
rea	Interferon Signaling	REAC:913531	196	4.13e-10	12	9
rea	Interferon alpha/beta signaling	REAC:909733	69	8.59e-14	11	6
rea	O2/CO2 exchange in erythrocytes	REAC:1480926	13	3.16e-03	3	
rea	Erythrocytes take up carbon dioxide and release oxygen	REAC:1237044	13	3.16e-03	3	
rea	Erythrocytes take up oxygen and release carbon dioxide	REAC:1247673	9	9.38e-04	3	
rea	Cell Cycle	REAC:1640170	602	4.08e-08		20
rea	Cell Cycle, Mitotic	REAC:69278	496	1.19e-08		19
rea	M Phase	REAC:68886	302	6.00e-05		12
rea	Mitotic Prometaphase	REAC:68877	108	4.48e-06		9
rea	Resolution of Sister Chromatid Cohesion	REAC:2500257	100	4.19e-05		8
rea	Mitotic Metaphase and Anaphase	REAC:2555396	176	2.70e-02		7
rea	Mitotic Anaphase	REAC:68882	175	2.60e-02		7
rea	Separation of Sister Chromatids	REAC:2467813	164	1.72e-02		7
rea	RHO GTPase Effectors	REAC:195258	290	1.73e-02		9
rea	RHO GTPases Activate Formins	REAC:5663220	115	1.71e-03		7
rea	Polo-like kinase mediated events	REAC:156711	16	3.45e-02		3

source	term name<br>Regulatory motifs in DNA (TRANSFAC TFBS)	term ID	n. of term genes	corrected p-value	OURS	LONG_ET_AL
tf	Factor: ICSBP; motif: RAARTGAAACTG; match class: 0	TF:M00699_0	3727	8.70e-04	20	
tf	Factor: IRF-4; motif: KRAAMNGAAANYN; match class: 1	TF:M07323_1	3362	2.07e-02	17	
tf	Factor: IRF5; motif: CCGAAACCGAAACY; match class: 0	TF:M04016_0	787	7.41e-04	10	
tf	Factor: ISGF-3; motif: CAGTTTCWCTTTYCC; match class: 0	TF:M00258_0	1741	1.55e-06	17	
tf	Factor: IRF1; motif: NNNYASTTTCACTTTCNNTTT; match class: 0	TF:M07216_0	841	1.17e-02	9	
tf	Factor: IRF-7; motif: TNSGAAWNCGAAANTNNN; match class: 0	TF:M00453_0	5414	4.94e-03	23	
tf	Factor: IRF-7; motif: TNSGAAWNCGAAANTNNN; match class: 1	TF:M00453_1	816	7.01e-07	13	
